# Technology Activities and Cognitive Trajectories Among Community-Dwelling Older Adults: National Health and Aging Trends Study

**DOI:** 10.2196/77227

**Published:** 2025-11-25

**Authors:** Erh-Chi Hsu, Erin M Spaulding, Eric Jutkowitz

**Affiliations:** 1 School of Nursing, Johns Hopkins University, 525 N Wolfe St, Baltimore, MD, 21205, United States, 1 2066175816; 2 Digital Health Innovation Laboratory, Ciccarone Center for the Prevention of Cardiovascular Disease, Division of Cardiology, Department of Medicine, Johns Hopkins University School of Medicine, Baltimore, MD, United States; 3 Welch Center for Prevention, Epidemiology, and Clinical Research, Johns Hopkins Bloomberg School of Public Health, Baltimore, MD, United States; 4 School of Public Health, University of Minnesota, Minneapolis, MN, United States

**Keywords:** aging, cognitive function, technology use, digital activities, NHATS, National Health and Aging Trends Study

## Abstract

**Background:**

While the positive effects of digital technology on cognitive function are established, the specific impacts of different types of technology activities on distinct cognitive domains remain underexplored.

**Objective:**

This study aimed to examine the associations between transitions into and out of various technology activities and trajectories of cognitive domains among community-dwelling older adults without dementia.

**Method:**

Data were drawn from 5566 community-dwelling older adults without dementia who participated in the National Health and Aging Trends Study from 2015 to 2022. Technology activities assessed included online shopping, banking, medication refills, social media use, and checking health conditions online. The cognitive domains measured were episodic memory, executive function, and orientation. Asymmetric effects models were used to analyze the associations between technology activity transitions and cognitive outcomes, adjusting for demographic, socioeconomic, and health-related covariates. Lagged models were applied for sensitivity analysis.

**Results:**

In the asymmetric effects models, the onset of online shopping (*β*=.046, *P*=.02), medication refills (*β*=.073, *P*<.001), and social media use (*β*=.065, *P*=.01) was associated with improved episodic memory. The cessation of online shopping was associated with faster episodic memory decline (*β*=−.023, *P*=.047). In contrast, the cessation of online banking (*β*=−.078, *P*=.01) and social media use (*β*=−.066, *P*=.003) was associated with decreased episodic memory. The initiation of instrumental, social, and health-related technology activities was associated with slower cognitive decline in orientation. The lagged models further emphasized the effects of stopping online banking and starting online medication refills in relation to episodic memory, as well as the positive associations between online shopping and social media use and orientation. All significant effects were of small magnitude.

**Conclusions:**

Combining findings from the main and sensitivity analyses, results suggest that interventions designed to support episodic memory in older adults should emphasize promoting the use of online medication refill services and sustaining engagement with online banking, particularly among those who have already established these habits. To support orientation, strategies should focus on facilitating adoption of online shopping and social media use, helping older adults become comfortable navigating these platforms. Future trials are needed to assess the clinical relevance of targeted interventions for specific cognitive domains, to promote the initiation and maintenance of digital activities to help mitigate domain-specific cognitive decline in aging populations.

## Introduction

As technology use becomes increasingly prevalent among older adults, its potential impact on cognitive health warrants attention. Between 2018 and 2021, smartphone ownership among individuals aged 65 years and older in the United States increased from 46% to 61%, outpacing growth in younger age groups [1]. Similarly, tablet ownership increased from 6% to 44%, and social media use surged from 16% to 45% over the past decade [1]. This rise in digital engagement holds the potential to influence various aspects of older adults’ lives, including their cognitive function. Research indicates that digital technology use is associated with higher social well-being and lower depressive symptoms in older adults [2,3]. In addition, technology can aid in managing instrumental activities of daily living (IADLs), which are crucial for maintaining independence (eg, grocery shopping online) [4]. While cognitive health is crucial for preserving autonomy and quality of life in aging populations, and technology appears to support these goals, the specific relationship between technology use and cognitive function remains understudied, particularly among older adults without dementia.

Cognitive health, encompassing domains such as episodic memory, executive function, and orientation, is a crucial predictor of health outcomes, quality of life, and cognitive trajectories in older adults [5-10]. Declines in cognitive function are associated with diminished quality of life [11] and greater financial strain on individuals and their families [12]. In addition, cognitive health is closely linked to the ability to perform daily activities, including those involving digital technologies. From a neurobiological standpoint, cognitive functions are supported by intricate brain networks. The hippocampus is essential for episodic memory, allowing flexible retrieval of information and aiding social interactions by integrating memory with context [13,14]. The prefrontal cortex manages goal-directed actions through top-down control, coordinating activity across different brain regions, which underlies executive function [15]. Moreover, spatial and temporal orientation involve the temporoparietal junction, a region crucial for navigating through time and space [16].

Many digital tasks, such as managing medications online, navigating shopping sites, or using social media, require a combination of these cognitive skills. Performing these activities involves remembering steps, following instructions, organizing information, engaging with digital interfaces, and maintaining awareness of dates and times. Behaviorally, cognitive decline is strongly associated with a reduced ability to perform basic and IADLs [17]. Recent research using network analysis has demonstrated strong associations between nondigital IADLs (eg, banking and shopping) and specific cognitive domains such as memory and orientation [18]. These findings underscore the importance of studying digital equivalents of such tasks.

Most studies have focused on general internet use and global cognitive functioning, without examining the nuanced impact of transitions into and out of specific technology activities on different cognitive domains. For example, a recent study using the National Health and Aging Trends Study (NHATS) indicated that cessation of internet use predicted declines in episodic memory, executive function, and orientation [19]. However, that study treated internet use as a single broad category, without differentiating between specific online activities. Similarly, while Li et al [18] found connections between instrumental activities and specific cognitive domains, it remains unclear whether these relationships translate to their digital counterparts. Other research using the Health and Retirement Study has examined different types of internet use—such as social, informational, and instrumental purposes—and found associations with better global cognitive scores [20]. Together, these studies highlight a key gap: although digital engagement appears to influence cognitive health, it is not yet clear which types of technology activities are related to specific cognitive domains. Addressing this gap is crucial for informing resource allocation and intervention design, ensuring that limited resources are used effectively and that interventions target the cognitive domains most likely to benefit from technology use. This study aimed to address the following 2 research questions:

1. What are the associations between technology activities (ie, online shopping, banking, medication refills, social media use, and checking health information) and cognitive domains (ie, episodic memory, executive function, and orientation) among community-dwelling older adults without dementia?
2. Does the start and stop of using specific technology activities moderate the rate of cognitive decline over time in community-dwelling older adults without dementia?

## Methods

### Data Source and Study Population

This project involved a secondary quantitative data analysis using data from the NHATS collected between 2015 and 2022 (Rounds 5-12). NHATS, supported through a cooperative agreement with the National Institute on Aging (U01AG032947), is a nationally representative longitudinal study of Medicare beneficiaries aged 65 years and older [21]. Annual interviews have been conducted since 2011 (Round 1), with sample replenishment in 2011, 2015, and 2022 (Rounds 1, 5, and 12 [21]). A detailed cohort profile is available in Freedman and Kasper [21], published by the NHATS study team.

The sample consisted of community-dwelling individuals aged 65 years or older without a diagnosis of dementia or mild cognitive impairment at baseline [5]. The original analytic sample included 5596 community-dwelling individuals aged 65 years and older without dementia. We identified 30 out of 5596 participants who were classified as having no dementia but lacked cognitive test scores. After consulting with the NHATS team, they indicated that these participants, or their proxy respondents, refused to complete the cognition test. In such cases, NHATS relied solely on self-reports of dementia instead of the tests. Since cognition scores were the main outcome measure, the level of missingness was minimal (0.5%), and these cases were excluded from the analysis. The final sample size in 2015 (Round 5) included 5566 older adults, with 31,038 observations from 2015 to 2022 (Rounds 5-12). The median number of follow-up observations per round was 3631.

### Measures

#### Independent Variables

Five technology activities were the primary variables of interest in this study: (1) online shopping, (2) online banking, (3) online medication refills, (4) visiting social networking sites, and (5) checking health information online. These questions were asked across all 8 rounds of data collection. Each activity was measured using dichotomous (yes or no) questions, as phrased in Table 1.

Instead of using raw binary variables for technology activities, we created new variables to capture cumulative transitions into and out of each activity over time. Specifically, a Cumulative Transition In variable was incremented by one each time an individual’s response changed from “no=0” to “yes=1” for a given technology activity. Similarly, a Cumulative Transition Out variable was incremented by one each time a response shifted from “yes=1” to “no=0.” This approach allowed us to focus on the effects of transitions (onset or cessation of use) rather than the mere status of technology use. This within-person asymmetric effect method has been used in previous studies, such as Kim and Han and Ghaiumy Anaraky et al [19,22]. Multimedia Appendix 1 shows how cumulative transitions were calculated for online shopping as an example.

**Table 1.** Exposure measurements.

Topics	Questions
Online shopping	In the last month, have you gone on the internet or online to shop for groceries or personal items? If needed: by personal items, we mean things such as toiletries that you can buy at the grocery or drug store.
Online banking	In the last month, have you gone on the internet or online to pay bills or do banking?
Online medication refills	In the last month, have you gone on the internet or online to order or refill prescriptions?
Visit social network sites	In the last month, have you gone on the internet or online to visit social network sites? If needed, these include sites where you can keep in touch with friends, such as Facebook or LinkedIn.
Check health information online	In the last year, have you gone on the internet or online to get information about your health conditions?

#### Dependent Variables

The primary outcome, cognitive function, was assessed across 3 domains. Episodic memory, the ability to recall personal experiences that are tied to specific times and locations, was scored from 0 to 20 based on immediate and delayed recall of 10 words. Executive function, which involves decision-making and problem-solving, was scored from 0 to 5 through the clock-drawing test. Orientation, the ability to recognize one’s identity, spatial context, and time, was scored from 0 to 8 based on knowledge of the current location, time, date, and the names of the president or vice president. To enable comparisons across these differently scaled domains, each score was standardized by converting it to a *z* score (original score minus mean, divided by SD).

#### Covariates

Covariates included survey year (2015‐2022), sex (female or male), age at baseline (continuous), race (non-Hispanic White, non-Hispanic Black, non-Hispanic Asian American and Pacific Islander, or Hispanic), education (no college degree, college degree, or beyond), living arrangement (alone or with others), number of devices owned (cell phone, computer, or tablet; ranging 0‐3), rurality (metropolitan or nonmetropolitan), number of activities of daily living difficulties (discrete), number of IADL difficulties (discrete), self-rated health (poor, fair, good, very good, and excellent). Covariates were selected based on established associations with cognitive function in older adults. Demographic factors (sex, age, race, education, and living arrangement) were included as supported by Ghaiumy Anaraky et al [19]. Device ownership reflects digital access and has been linked to slower cognitive decline [23]. Activities of daily living and IADLs were included due to their strong connections to cognitive health [18,24]. Rurality and self-rated health are known predictors of cognitive outcomes [25,26].

### Weighting

In our study, we applied analytic weights using the weighting variables from the sample person file [27]. Since the 2015 cohort was chosen as the foundation of our target population, we tackled the problem of multiple (repeated) observations through a multilevel modeling strategy that incorporated the NHATS sample design. The weights for each round were used to account for differential probabilities of selection and nonresponse, as well as for clustering and stratification variables. Adhering to the guidelines and code detailed in NHATS Technical Paper 23 [28], we used round-specific analytic weights and developed a new weight variable to represent individual-level weights within the multilevel models [28].

### Statistical Analysis

We conducted generalized linear mixed-effects models separately for the 3 cognitive domains, both unadjusted and adjusted for covariates. The predictors were various types of technology activities, and the outcomes were the different domains of cognition, including episodic memory, executive function, and orientation.

General linear mixed-effects models are particularly suited for analyzing longitudinal or hierarchical data, where repeated measurements are nested within individuals. Given the nature of cognitive function may change over time due to aging and may also be influenced by exposure to different types of technology, the use of general linear mixed-effects models provided an appropriate statistical framework for this analysis.

Two models were specified: the main effect model (Model A), which assessed the association between transitions in technology use and cognitive function, and the moderation effect model (Model B), which indicated the effects of technology use on the rate of change in cognitive functioning. For example, the main effect model (Model A) for episodic memory and online shopping was specified as follows:


$$Episodic\;Memory_{ij}\;=\;\beta_{0}\;+\;\beta_{1}Transition\;In\;Online\;Shopping_{ij}\;+\;\beta_{2}Transition\;Out\;Online\;Shopping_{ij}\;+\;\beta_{3}Year_{ij}\;+\;\beta_{4}Sex_{i}\;\;+\;\beta_{5}Baseline\;Age_{i}\;+\;\beta_{6}Race_{i}\;+\;\beta_{7}Education_{i}\;+\;\beta_{8}Living\;Arrangement_{i}+\;\beta_{9}Device\;Ownership_{ij}\;+\;\beta_{10}Rurality_{i}\;\;+\;\beta_{11}ADL_{ij}\;+\;\beta_{12}IADL_{ij}+\;\beta_{13}Self\;Rated\;Health_{ij}+\;u_{oi}+\;\varepsilon_{ij}$$

The moderation effects model (Model B) added interaction terms for transitions and time, as follows:


$$Episodic\;Memory_{ij}\;=\;\beta_{0}+\;\beta_{1}Transition\;In\;Online\;Shopping_{ij}\;+\;\beta_{2}Transition\;Out\;Online\;Shopping_{ij}+\beta_{3}Year_{ij}\;\;+\;\beta_{4}\left(Transition\;In\;Online\;Shopping_{ij}\times Year_{ij}\right)\;+\;\beta_{5}\left(Transition\;Out\;Online\;Shopping_{ij}\times Year_{ij}\right)+\;\beta_{6}Sex_{i}\;\;+\;\beta_{7}Baseline\;Age_{i}+\beta_{8}Race_{i}\;+\;\beta_{9}Education_{i}\;+\;\beta_{10}Living\;Arrangement_{i}+\beta_{11}Device\;Ownership_{ij}+\beta_{12}Rurality_{i}\;\;+\;\beta_{13}ADL_{ij}+\beta_{14}IADL_{ij}+\;\beta_{15}Self\;Rated\;Health_{ij}+u_{oi}+\varepsilon_{ij}$$

The index *i* represents the individual sample ID, and *j* denotes the time point (ie, *j*=2015, 2016, ..., 2022).

We first estimated whether the start (transition in) and stop (transition out) of technology activities were associated with cognitive functioning at a given time (Model A). We then added an interaction term between transitions and time to explore whether starting or stopping technology activities could change the rate of cognitive decline over time (Model B).

Since we evaluated multiple outcomes, we applied the Benjamini-Hochberg false discovery rate correction to reduce the risk of type I errors [29]. To minimize the possibility of a reverse causal relationship between digital activities and cognitive domains, we conducted sensitivity analyses using 1-year lagged data for both Model A and Model B to ensure the associations hold over time.

### Ethical Considerations

This study was exempted by the Johns Hopkins Medicine Institutional Review Board (IRB00460714), where informed consent is not required. The NHATS public use data is de-identified.

## Results

### Characteristics of the Study Sample at Baseline

The study sample characteristics at baseline are provided in Table 2. At baseline, the study sample (N=5566; representing an estimated 32,929,570 older adults living in communities in the United States) had an average age of 76.48 (SD 7.04) years and no dementia. The sample was predominantly female (3190, 57.31%), with well-distributed racial and ethnic groups. Most participants had no college degree (3738, 67.16%), lived with someone else (3803, 68.33%), and resided in metropolitan areas (4476, 80.42%). Furthermore, most participants owned one or more digital devices (eg, cell phone, computer, or tablet), and around a quarter of them had performed some technology activities in the past month.

**Table 2.** Study sample characteristics at baseline (N=5566).

Characteristic	Value
Age (years), mean (SD)	76.48 (7.04)
Episodic memory (0‐20), mean (SD)	9.13 (2.93)
Executive function (0‐5), mean (SD)	3.91 (0.93)
Orientation (0‐8), mean (SD)	6.88 (1.24)
Sex, n (%)	
Male	2376 (42.69)
Female	3190 (57.31)
Race and ethnicity, n (%)	
Non-Hispanic White	4005 (71.95)
Non-Hispanic Black	1021 (18.34)
Non-Hispanic AAPI^a^	145 (2.66)
Hispanic	274 (4.92)
Missing	121 (2.17)
Number of device ownership (cell phone, computer, and tablet), n (%)	
0	376 (6.76)
1	1421 (25.53)
2	1949 (35.02)
3	1820 (32.70)
Education, n (%)	
No college degree	3738 (67.16)
College degree or beyond	1828 (32.84)
Living arrangement, n (%)	
Alone	1763 (31.67)
Living with someone	3803 (68.33)
Rurality, n (%)	
Metropolitan	4476 (80.42)
Non-metropolitan	1090 (19.58)
Number of difficulties in ADL^b^, mean (SD)	0.63 (1.27)
Number of difficulties in IADL^c^, mean (SD)	0.42 (0.86)
Self-rated health, n (%)	
Excellent	724 (13.01)
Very good	1717 (30.86)
Good	1917 (34.45)
Fair	975 (17.52)
Poor	231 (4.15)
Technology activities, n (%)	
Online shopping	1129 (20.3)
Online banking	1553 (27.9)
Online medication refills	665 (11.9)
Visit social network sites	1414 (25.4)
Checking health information online	1198 (21.5)

a AAPI: Asian American and Pacific Islander.

b ADL: activities of daily living.

c IADL: instrumental activities of daily living.

### The Associations Between Technology Activities and Cognitive Domains

Table 3 shows the beta coefficients for the initiation and cessation of each technology activity across various cognitive domains. These findings stem from Model A and address research question 1—the associations between technology activities (online shopping, banking, medication refills, social media use, and checking health information) and cognitive domains (episodic memory, executive function, and orientation). *P* values are marked after the false discovery rate correction using the Benjamini-Hochberg method. The *P* values before and after adjustment are provided in Multimedia Appendix 2.

Transitions into online shopping were associated with an increase in the score of episodic memory at a given wave (𝛽=.046, *P*=.02; 95% CI 0.013-0.080; Model A), as well as an increase in the score of executive function (𝛽=.041, *P*=.043; 95% CI 0.007-0.075; Model A) and orientation at a given wave (𝛽=.091, *P*<.001; 95% CI 0.055-0.126; Model A).

Starting online banking was associated with an increase in executive function at a given wave (𝛽=.061, *P*=.043; 95% CI 0.010-0.112; Model A) and orientation (𝛽=.077, *P*=.004; 95% CI 0.032-0.123; Model A). Stopping online banking was associated with decreasing scores of episodic memory (𝛽=−.078, *P*=.01; 95% CI −0.129 to −0.026; Model A).

Transitions into online medication refills were associated with increasing episodic memory (𝛽=.073, *P*<.001; 95% CI 0.038-0.109; Model A) and increasing orientation at a given wave (𝛽=.097, *P*<.001; 95% CI 0.054-0.140; Model A).

Starting to visit social networking sites online was associated with increasing episodic memory (𝛽=.065, *P*=.01; 95% CI 0.022-0.109; Model A) and orientation at a given wave (𝛽=.080, *P*<.001; 95% CI 0.041-0.119; Model A). Stopping visits to social network sites online was associated with decreasing episodic memory (𝛽=−.066, *P*=.004; 95% CI −0.105 to −0.028; Model A). Transitions into checking health information online were associated with increasing orientation (𝛽=.098, *P*<.001; 95% CI 0.068-0.127; Model A) at a given wave.

Among all the technology activities examined, online shopping and online banking showed the widest impact across various areas of cognitive function. Combining our main findings with results from lagged models in sensitivity analyses (Multimedia Appendix 3), older adults who started using online shopping and social media were related to better orientation; those who began managing prescriptions digitally showed cognitive benefits in episodic memory; and those who stopped using online banking were linked to a decline in episodic memory. Notably, the effects were statistically significant but small in magnitude.

**Table 3.** Effects of starting and stopping technology activities on cognitive domains (Model A).

Technology activity	Episodic memory *z* score, β (95% CI)		Executive function *z* score, β (95% CI)		Orientation *z* score, β (95% CI)	
	Start	Stop	Start	Stop	Start	Stop
Online shopping	0.046^a^(0.013 to 0.080)	0.019 (−0.020 to 0.058)	0.041^b^ (0.007 to 0.075)	0.004 (−0.062 to 0.069)	0.091^c^ (0.055 to 0.126)	0.010 (−0.026 to 0.046)
Online banking	0.049 (−0.003 to 0.100)	−0.078^a^ (−0.129 to 0.026)	0.061^b^ (0.010 to 0.112)	−0.059^b^ (−0.117 to 0.000)	0.077^a^ (0.032 to 0.123)	−0.062 (−0.126 to 0.003)
Medication refills	0.073^c^ (0.038 to 0.109)	−0.014 (−0.068 to 0.040)	0.026 (−0.014 to 0.067)	0.011 (−0.038 to 0.059)	0.097^c^ (0.054 to 0.140)	−0.007 (−0.055 to 0.040)
Social media	0.065^a^ (0.022 to 0.109)	−0.066^a^ (−0.105 to 0.028)	0.050 (−0.002 to 0.101)	−0.045 (−0.094 to 0.004)	0.080^c^ (0.041 to 0.119)	−0.024 (−0.068 to 0.021)
Check health information	0.047 (0.005 to 0.089)	0.027 (−0.014 to 0.067)	−0.011 (−0.054 to 0.031)	0.060^c^ (0.032 to 0.088)	0.098^c^ (0.068 to 0.127)	0.048^b^ (0.010 to 0.085)

a *P*<.01.

b *P*<.05.

c *P*<.001.

### Effects of Technology Activities on the Rate of Cognitive Decline

In Model B, we added an interaction term between transitions of technology activities and time to assess their effect on the rate of cognitive decline. Table 4 shows the beta coefficients for the interaction terms between shifts in technology activities and time, reflecting the rate of cognitive decline. A positive value indicates a reduction in cognitive decline, while a negative score suggests an increase in cognitive decline.

Stopping online shopping was associated with a faster rate of cognitive decline in episodic memory (𝛽=−.023, *P=*.047; 95% CI −0.041 to−0.005; Model B). The transition into online shopping was associated with a slower cognitive decline in the orientation domain (𝛽=.049, *P*<.001; 95% CI 0.035-0.063; Model B). The onset of online banking was associated with a mitigated rate of orientation decline (𝛽=.049, *P*<.001; 95% CI 0.035-0.063; Model B). The onset of online medication refill was associated with a slower orientation decline (𝛽=.052, *P*<.001; 95% CI 0.036-0.068; Model B). The onset of social media use was associated with a slower decline in the orientation domain (𝛽=.039, *P*<.001; 95% CI 0.025-0.053; Model B). The transition into checking health information online was associated with a mitigated rate of orientation decline (𝛽=.036, *P*<.001; 95% CI 0.022-0.051; Model B). When incorporating findings from lagged models in sensitivity analyses (Multimedia Appendix 3), regarding the interaction with time, beginning to use any of the studied digital technologies was linked to significantly better but small preservation of orientation.

The summary of all models, stratified by technology activities, is provided in Multimedia Appendix 4. Several noteworthy confounders emerged in the analysis. A higher number of device owners was consistently associated with better cognitive functioning across all technology activities. Similarly, individuals with higher educational attainment (college degree or beyond) demonstrated superior performance across all cognitive domains. Females exhibited stronger effects specifically in episodic memory, regardless of the technology activity.

**Table 4.** The interaction coefficients of starting and stopping technology activities on cognitive domains (Model B).

Technology activity	Episodic memory *z* score, β (95% CI)		Executive function *z* score, β (95% CI)		Orientation *z* score, β (95% CI)	
	Start	Stop	Start	Stop	Start	Stop
Online shopping	0.014 (0.000 to 0.027)	−0.023 ^a^(−0.041 to 0.005)	0.004 (−0.011 to 0.018)	−0.000 (−0.016 to 0.016)	0.049^b^ (0.035 to 0.063)	−0.011 (−0.026 to 0.004)
Online banking	0.016 (−0.001 to 0.032)	−0.010 (−0.030 to 0.011)	0.020 (−0.003 to 0.043)	−0.017 (−0.045 to 0.010)	0.044^b^ (0.021 to 0.067)	0.005 (−0.016 to 0.026)
Medication refills	−0.001 (−0.017 to 0.015)	−0.012 (−0.037 to 0.012)	0.015 (−0.006 to 0.037)	−0.013 (−0.042 to 0.016)	0.052^b^ (0.036 to 0.068)	0.005 (−0.012 to 0.021)
Social media	0.010 (−0.005 to 0.026)	−0.022 (−0.040 to 0.004)	−0.008 (−0.026 to 0.009)	0.008 (−0.014 to 0.030)	0.039^b^ (0.025 to 0.053)	−0.002 (−0.018 to 0.015)
Check health information	0.001 (−0.014 to 0.015)	−0.006 (−0.021 to 0.009)	0.004 (−0.014 to 0.022)	0.002 (−0.018 to 0.022)	0.036^b^ (0.022 to 0.051)	0.020^c^ (0.005 to 0.034)

a *P*<.05.

b *P*<.001.

c *P*<.01.

## Discussion

### Principal Findings

This study highlights the distinct effects of technology use on specific cognitive domains among older adults. Compared with other digital activities, the use of online shopping and online banking services showed the most comprehensive influence on the 3 cognitive domains. Similar to findings from a previous study, online shopping and banking involve a series of actions such as memorizing accounts and passwords, thinking about recipes and corresponding products to purchase, or financial plans to arrange, and remaining oriented to dates, time, and their own names to process transactions [18,30].

Older adults who began using online shopping and social media showed a small improvement in orientation. These activities may enhance spatial and temporal awareness by requiring users to follow delivery schedules, navigate websites, and engage with time-stamped social content. Starting to manage prescriptions online was linked to better episodic memory. This task likely activates memory systems through routine recall of medications and schedules, reinforcing memory through repeated use and the integration of new and existing information. In contrast, stopping online banking was associated with a decline in episodic memory. Online banking involves complex memory tasks such as recalling passwords and managing finances. Disengaging from such cognitively demanding activities may reduce mental stimulation, contributing to memory decline. However, all observed effects were small in magnitude, and their clinical significance remains uncertain. Future trials are needed to evaluate the clinical relevance of targeted digital engagement interventions on specific cognitive domains.

### Limitations

This study used observational data, which introduces the possibility of reverse causation. It is plausible that older adults with lower cognitive performance were less likely to adopt or continue using technology. However, findings from Choi et al [31], using a cross-lagged panel analysis of NHATS data, suggest that technology use predicts cognitive improvements over time, particularly in episodic memory and executive function. While episodic memory also predicted subsequent technology use, executive function did not, and orientation was not assessed in their study [31]. Similarly, Hartanto et al [32], using data from the Midlife in the United States study, found that computer use significantly predicted positive changes in executive function. Their analysis showed consistent associations between computer use and improvements across all executive function tasks, though no significant effect was observed for episodic memory. Collectively, these findings support a stronger directional relationship from technology use to cognitive function, although a bidirectional relationship cannot be ruled out.

Some limitations stemmed from the instrument used by NHATS. For example, the question about social media use simply asked whether the participant had gone online to visit social network sites (eg, Facebook and LinkedIn) in the last month. The specific platform, content on the sites, and the level of engagement were not asked. We recognize the heterogeneity of social media and the potential varied associations with cognitive function. In addition, digital activity questions were designed to help participants recall their technology use behaviors over the past month or year, depending on the activity. This study only assessed changes in technology use and cognition over 8 years, with the smallest time unit being a year. Thus, one instance of use within a given year was coded as “yes,” regardless of frequency. We acknowledge that this annual resolution limits our ability to capture short-term fluctuations in digital engagement or the precise timing of technology use relative to cognitive assessments.

Attrition in this study occurred through 3 primary mechanisms: death, nonresponse, and transitions from community to institutional settings. While the NHATS cohort maintained high response rates (>90%) with relatively low attrition per round [21], we used weighted analyses to account for potential attrition bias. Analysis of NHATS data from 2011 to 2018 revealed that 86.2% of older adults remained in community settings, 9% transitioned to residential care, and 4.9% to nursing homes [33]. Although only a small proportion of older adults changed settings, these transitions predominantly occurred among older adults with greater functional and cognitive limitations, potentially limiting our ability to capture technology access and use patterns among this more vulnerable subgroup.

Another limitation concerns the last few years of the study period, which coincided with the COVID-19 pandemic. Social isolation during this time was particularly acute for older adults [34]. Evidence from NHATS data shows that older adults who reduced their use of emails, texts, and phone calls during the pandemic, especially those with lower socioeconomic status, showed a limited increase in video calls to stay connected with family and friends [35]. The pandemic’s potential impact on technology use trends and its subsequent influence on cognitive function remains unclear, introducing uncertainty to the study’s results.

In considering generalizability, it is important to note that wired high-speed internet access remains more prevalent in urban than rural areas—available in 76% of noninstitutional households in urban settings compared with 62% in rural areas as of 2023 in the United States [36]. While our findings highlight the potential cognitive benefits of certain digital activities, real-world implementation may be limited by disparities in local infrastructure and household financial capacity. Without reliable internet access or the resources to obtain and maintain digital devices, many older adults, particularly in underserved areas, may be unable to engage in these beneficial activities.

### Comparison With Previous Work

The observed association between social media use and cognitive function aligns with previous research. For example, Byrne and Ghaiumy Anaraky [37] found that greater use of social technology was linked to enhanced cognitive performance among socially isolated older adults, though their outcome measure was a global cognitive score rather than disaggregated domains. Anaraky et al [19] reported that stopping the use of computers, tablets, the internet, texts, and emails predicted declines in episodic memory, executive function, and orientation. Similarly, Kim and Han [22] reported that initiating internet use was associated with improved cognitive functioning and a slower rate of cognitive decline over time, while ceasing internet use was linked to worse cognitive outcomes and accelerated decline. These findings align with this study’s results, which show that both initiating and maintaining technology use are crucial for cognitive health. Our study provides more granular insights, demonstrating the unique effects of various technology activities on episodic memory, executive function, and orientation.

### Conclusions

Our findings suggest that interventions aiming to maintain episodic memory in older adults should focus on encouraging the adoption of online medication refill services and preventing disengagement from online banking, particularly among those already accustomed to these activities. To preserve orientation, efforts should prioritize supporting older adults in initiating and maintaining online shopping and social media use. More broadly, all types of digital engagement examined in this study—including instrumental, social, and informational activities—were associated with a slower decline in orientation over time. Teaching older adults new technology skills and providing resources to prevent disengagement from technology activities may help preserve cognitive health. Furthermore, when designing technology-based interventions, researchers can use these findings to target specific cognitive domains with appropriate technology activities. By tailoring interventions to match the cognitive benefits of different activities, resources can be allocated more effectively to maximize their impact on cognitive well-being in older adults.

## Supplementary material

10.2196/77227 Multimedia Appendix 1 An example of data structure.

10.2196/77227 Multimedia Appendix 2 Raw and false discovery rate (FDR)-adjusted *P* values for Models A and B.

10.2196/77227 Multimedia Appendix 3 Sensitivity analyses using lagged models.

10.2196/77227 Multimedia Appendix 4 Asymmetric random-effects models of technology activities and cognitive domains (*z* score).

## Abbreviations

IADL: instrumental activities of daily living
NHATS: National Health and Aging Trends Study
