# Supplementary material for: Technology Activities and Cognitive Trajectories Among Community-Dwelling Older Adults: National Health and Aging Trends Study
Source: JMIR Aging. 2025 Nov 25;8:e77227. doi: 10.2196/77227 (PMC12646554; doi:10.2196/77227)
Supplement: Multimedia Appendix 1 [file aging-v8-e77227-s001.docx]

| **Sample ID** | **Year** | **Episodic Memory** | **Executive Function** | **Orientation** | **Online Shopping**  **(Yes/No)** | **Cumulative Transition In**  **Online Shopping** | **Cumulative Transition Out Online Shopping** | **Covariates** |
| --- | --- | --- | --- | --- | --- | --- | --- | --- |
| **10000036** | 2015 | 9 | 3 | 8 | 0 | 0 | 0 |  |
| **10000036** | 2016 | 12 | 4 | 8 | 0 | 0 | 0 |  |
| **10000036** | 2017 | 10 | 4 | 8 | 1 | 1 | 0 |  |
| **10000036** | 2018 | 9 | 3 | 8 | 1 | 1 | 0 |  |
| **10000036** | 2019 | 6 | 3 | 6 | 1 | 1 | 0 |  |
| **10000036** | 2020 | 8 | 4 | 8 | 0 | 1 | 1 |  |
| **10000036** | 2021 | 8 | 3 | 6 | 1 | 2 | 1 |  |
| **10000036** | 2022 | 5 | 4 | 8 | 1 | 2 | 1 |  |
| **10000041** | 2015 | 1 | 3 | 5 | 0 | 0 | 0 |  |
| **10000041** | 2016 | 6 | 4 | 5 | 0 | 0 | 0 |  |
| **10000041** | 2017 | 2 | 3 | 5 | 0 | 0 | 0 |  |
| **10000041** | 2018 | 2 | 4 | 4 | 0 | 0 | 0 |  |
| **10000041** | 2019 | 6 | 5 | 5 | 0 | 0 | 0 |  |
| **10000041** | 2020 | 6 | 3 | 5 | 0 | 0 | 0 |  |
| **10000041** | 2021 | 3 | 3 | 6 | 0 | 0 | 0 |  |
| **10000041** | 2022 | 4 | 3 | 4 | 0 | 0 | 0 |  |
| … |  |  |  |  |  |  |  |  |
