# Supplementary material for: Technology Activities and Cognitive Trajectories Among Community-Dwelling Older Adults: National Health and Aging Trends Study
Source: JMIR Aging. 2025 Nov 25;8:e77227. doi: 10.2196/77227 (PMC12646554; doi:10.2196/77227)
Supplement: Multimedia Appendix 2 [file aging-v8-e77227-s002.docx]

**Raw and FDR-Adjusted P-Values for Effects of Start/Stop Technology Activities on Cognitive Domains (Model A)**

| Exposure Activity |  | Exposure | Outcome Domain | Raw p-value | FDR-adjusted p-value | Significant |
| --- | --- | --- | --- | --- | --- | --- |
| Online Shopping |  | Start | Episodic Memory | 0.01 | 0.03 | Yes |
| Online Shopping |  | Stop | Episodic Memory | 0.32 | 0.40 | No |
| Online Shopping |  | Start | Executive Function | 0.02 | 0.04 | Yes |
| Online Shopping |  | Stop | Executive Function | 0.99 | 0.99 | No |
| Online Shopping |  | Start | Orientation | 0.00 | 0.00 | Yes |
| Online Shopping |  | Stop | Orientation | 0.57 | 0.68 | No |
| Online Banking |  | Start | Episodic Memory | 0.06 | 0.09 | No |
| Online Banking |  | Stop | Episodic Memory | 0.00 | 0.00 | Yes |
| Online Banking |  | Start | Executive Function | 0.02 | 0.04 | Yes |
| Online Banking |  | Stop | Executive Function | 0.05 | 0.09 | No |
| Online Banking |  | Start | Orientation | 0.00 | 0.00 | Yes |
| Online Banking |  | Stop | Orientation | 0.06 | 0.09 | No |
| Medication Refills |  | Start | Episodic Memory | 0.00 | 0.00 | Yes |
| Medication Refills |  | Stop | Episodic Memory | 0.61 | 0.68 | No |
| Medication Refills |  | Start | Executive Function | 0.19 | 0.26 | No |
| Medication Refills |  | Stop | Executive Function | 0.66 | 0.71 | No |
| Medication Refills |  | Start | Orientation | 0.00 | 0.00 | Yes |
| Medication Refills |  | Stop | Orientation | 0.77 | 0.80 | No |
| Social Media |  | Start | Episodic Memory | 0.00 | 0.00 | Yes |
| Social Media |  | Stop | Episodic Memory | 0.00 | 0.00 | Yes |
| Social Media |  | Start | Executive Function | 0.06 | 0.09 | No |
| Social Media |  | Stop | Executive Function | 0.07 | 0.11 | No |
| Social Media |  | Start | Orientation | 0.00 | 0.00 | Yes |
| Social Media |  | Stop | Orientation | 0.29 | 0.38 | No |
| Check Health Info |  | Start | Episodic Memory | 0.03 | 0.06 | No |
| Check Health Info |  | Stop | Episodic Memory | 0.19 | 0.26 | No |
| Check Health Info |  | Start | Executive Function | 0.59 | 0.68 | No |
| Check Health Info |  | Stop | Executive Function | 0.00 | 0.00 | Yes |
| Check Health Info |  | Start | Orientation | 0.00 | 0.00 | Yes |
| Check Health Info |  | Stop | Orientation | 0.01 | 0.03 | Yes |

**Raw and FDR-adjusted P-Values for Interaction Effects of Start/Stop Technology Activities on Cognitive Domains (Model B)**

| Exposure Activity |  | Exposure | Domain | Raw p-value | FDR-adjusted p-value | Significant |
| --- | --- | --- | --- | --- | --- | --- |
| Online Shopping |  | Start | Episodic Memory | 0.04 | 0.13 | No |
| Online Shopping |  | Stop | Episodic Memory | 0.01 | 0.04 | Yes |
| Online Shopping |  | Start | Executive Function | 0.60 | 0.78 | No |
| Online Shopping |  | Stop | Executive Function | 0.99 | 0.99 | No |
| Online Shopping |  | Start | Orientation | 0.00 | 0.00 | Yes |
| Online Shopping |  | Stop | Orientation | 0.15 | 0.38 | No |
| Online Banking |  | Start | Episodic Memory | 0.07 | 0.21 | No |
| Online Banking |  | Stop | Episodic Memory | 0.34 | 0.57 | No |
| Online Banking |  | Start | Executive Function | 0.08 | 0.22 | No |
| Online Banking |  | Stop | Executive Function | 0.21 | 0.42 | No |
| Online Banking |  | Start | Orientation | 0.00 | 0.00 | Yes |
| Online Banking |  | Stop | Orientation | 0.65 | 0.78 | No |
| Medication Refills |  | Start | Episodic Memory | 0.91 | 0.97 | No |
| Medication Refills |  | Stop | Episodic Memory | 0.31 | 0.57 | No |
| Medication Refills |  | Start | Executive Function | 0.17 | 0.39 | No |
| Medication Refills |  | Stop | Executive Function | 0.36 | 0.57 | No |
| Medication Refills |  | Start | Orientation | 0.00 | 0.00 | Yes |
| Medication Refills |  | Stop | Orientation | 0.58 | 0.78 | No |
| Social Media |  | Start | Episodic Memory | 0.18 | 0.39 | No |
| Social Media |  | Stop | Episodic Memory | 0.02 | 0.08 | No |
| Social Media |  | Start | Executive Function | 0.33 | 0.57 | No |
| Social Media |  | Stop | Executive Function | 0.48 | 0.69 | No |
| Social Media |  | Start | Orientation | 0.00 | 0.00 | Yes |
| Social Media |  | Stop | Orientation | 0.85 | 0.94 | No |
| Check Health Info |  | Start | Episodic Memory | 0.94 | 0.97 | No |
| Check Health Info |  | Stop | Episodic Memory | 0.42 | 0.63 | No |
| Check Health Info |  | Start | Executive Function | 0.65 | 0.78 | No |
| Check Health Info |  | Stop | Executive Function | 0.84 | 0.94 | No |
| Check Health Info |  | Start | Orientation | 0.00 | 0.00 | Yes |
| Check Health Info |  | Stop | Orientation | 0.01 | 0.04 | Yes |
