# Supplementary material for: Technology Activities and Cognitive Trajectories Among Community-Dwelling Older Adults: National Health and Aging Trends Study
Source: JMIR Aging. 2025 Nov 25;8:e77227. doi: 10.2196/77227 (PMC12646554; doi:10.2196/77227)
Supplement: Multimedia Appendix 3 [file aging-v8-e77227-s003.docx]

**The Effects of Start/Stop Technology Activities on Cognitive Domains (Lagged Model A)**

|  | **Episodic Memory Z-Score** | | **Executive Function Z-Score** | | **Orientation Z-Score** | |
| --- | --- | --- | --- | --- | --- | --- |
|  | Start | Stop | Start | Stop | Start | Stop |
| **Online Shopping** | 0.009 (-0.041, 0.060) | 0.034 (-0.020, 0.089) | 0.016 (-0.038, 0.070) | 0.053 (-0.005, 0.111) | 0.103 *** (0.058, 0.148) | -0.047 (-0.096, 0.001) |
| **Online Banking** | 0.020 (-0.056, 0.095) | -0.095 * (-0.174, -0.016) | 0.004 (-0.078, 0.086) | -0.003 (-0.088, 0.081) | 0.006 (-0.062, 0.074) | -0.006 (-0.077, 0.065) |
| **Medication Refills** | 0.080 ** (0.022, 0.139) | -0.020 (-0.087, 0.048) | -0.019 (-0.082, 0.044) | -0.010 (-0.083, 0.063) | 0.018 (-0.035, 0.070) | 0.000 (-0.060, 0.061) |
| **Social Media** | 0.045 (-0.013, 0.104) | -0.018 (-0.078, 0.041) | -0.010 (-0.073, 0.053) | 0.013 (-0.051, 0.076) | 0.108 *** (0.055, 0.161) | -0.038 (-0.091, 0.015) |
| **Check Health Info** | 0.070 ** (0.018, 0.123) | -0.011 (-0.064, 0.042) | 0.009 (-0.048, 0.065) | 0.001 (-0.055, 0.058) | 0.030 (-0.016, 0.077) | 0.047 (-0.000, 0.095) |
| Note: 95% CIs in brackets **p*<0.05, ***p*<0.01, ****p*<0.001 | | | | | | |

**The Interaction Coefficients of Start/Stop Technology Activities on Cognitive Domains (Lagged Model B)**

|  | **Episodic Memory Z-Score** | | **Executive Function Z-Score** | | **Orientation Z-Score** | |
| --- | --- | --- | --- | --- | --- | --- |
|  | Start | Stop | Start | Stop | Start | Stop |
| **Online Shopping** | -0.007 (-0.035, 0.021) | -0.013 (-0.044, 0.018) | -0.023 (-0.054, 0.009) | -0.014 (-0.049, 0.022) | 0.037 ** (0.012, 0.062) | 0.042 ** (0.015, 0.070) |
| **Online Banking** | -0.031 (-0.075, 0.014) | 0.011 (-0.035, 0.057) | -0.049 (-0.099, 0.002) | 0.026 (-0.026, 0.079) | 0.097 *** (0.057, 0.138) | 0.001 (-0.041, 0.042) |
| **Medication Refills** | -0.004 (-0.039, 0.030) | -0.005 (-0.045, 0.035) | -0.016 (-0.055, 0.023) | -0.030 (-0.076, 0.015) | 0.067 *** (0.036, 0.098) | 0.039 * (0.003, 0.075) |
| **Social Media** | -0.019 (-0.053, 0.015) | -0.002 (-0.038, 0.033) | -0.023 (-0.062, 0.016) | -0.026 (-0.066, 0.014) | 0.054 *** (0.023, 0.084) | 0.042 * (0.010, 0.074) |
| **Check Health Info** | -0.027 (-0.056, 0.002) | 0.005 (-0.025, 0.035) | 0.024 (-0.010, 0.057) | -0.037 * (-0.071, -0.002) | 0.071 *** (0.045, 0.098) | 0.035 * (0.008, 0.062) |
| Note: 95% CIs in brackets **p*<0.05, ***p*<0.01, ****p*<0.001 | | | | | | |
