# Supplementary material for: Technology Activities and Cognitive Trajectories Among Community-Dwelling Older Adults: National Health and Aging Trends Study
Source: JMIR Aging. 2025 Nov 25;8:e77227. doi: 10.2196/77227 (PMC12646554; doi:10.2196/77227)
Supplement: Multimedia Appendix 4 [file aging-v8-e77227-s004.docx]

| **Asymmetric Random Effects Model of Online Shopping and Cognitive Domains (Z-Scores)** | | | | | | |
| --- | --- | --- | --- | --- | --- | --- |
|  | Episodic Memory  (Model A) | Episodic Memory  (Model B) | Executive Function  (Model A) | Executive Function  (Model B) | Orientation (Model A) | Orientation (Model B) |
| Intercept | 2.965*** | 2.960*** | 1.366*** | 1.371*** | 1.098*** | 1.156*** |
|  | [2.668,3.261] | [2.666,3.253] | [1.098,1.634] | [1.104,1.638] | [0.771,1.425] | [0.832,1.481] |
| Transition in Shopping | 0.046** | -0.057 | 0.041* | 0.007 | 0.091*** | -0.334*** |
|  | [0.013,0.080] | [-0.173,0.059] | [0.007,0.075] | [-0.118,0.133] | [0.055,0.126] | [-0.454,-0.214] |
| Transition out Shopping | 0.019 | 0.212** | 0.001 | -0.003 | 0.01 | 0.056 |
|  | [-0.020,0.058] | [0.071,0.352] | [-0.041,0.043] | [-0.139,0.134] | [-0.026,0.046] | [-0.071,0.183] |
| Time | -0.034*** | -0.034*** | -0.010** | -0.010** | -0.021*** | -0.022*** |
|  | [-0.041,-0.026] | [-0.041,-0.027] | [-0.017,-0.002] | [-0.017,-0.003] | [-0.028,-0.014] | [-0.029,-0.016] |
| Transition in Shopping * Time |  | 0.014* |  | 0.004 |  | 0.049*** |
|  |  | [0.000,0.027] |  | [-0.011,0.018] |  | [0.035,0.063] |
| Transition out Shopping * Time |  | -0.023* |  | 0 |  | -0.011 |
|  |  | [-0.041,-0.005] |  | [-0.016,0.016] |  | [-0.026,0.004] |
| Female | 0.299*** | 0.299*** | 0.047 | 0.047 | -0.014 | -0.016 |
|  | [0.257,0.342] | [0.256,0.341] | [-0.006,0.099] | [-0.006,0.099] | [-0.051,0.022] | [-0.053,0.021] |
| Number of Device Ownership (Cellphone, Computer, and Tablet) | | | | | | |
| 1 | 0.112** | 0.112** | 0.202*** | 0.202*** | 0.309*** | 0.309*** |
|  | [0.032,0.193] | [0.031,0.192] | [0.135,0.269] | [0.135,0.269] | [0.218,0.399] | [0.218,0.399] |
| 2 | 0.246*** | 0.244*** | 0.344*** | 0.345*** | 0.459*** | 0.460*** |
|  | [0.168,0.324] | [0.167,0.322] | [0.266,0.422] | [0.267,0.422] | [0.364,0.553] | [0.366,0.555] |
| 3 | 0.276*** | 0.274*** | 0.374*** | 0.375*** | 0.506*** | 0.512*** |
|  | [0.189,0.364] | [0.187,0.361] | [0.291,0.458] | [0.292,0.458] | [0.417,0.594] | [0.424,0.599] |
| Baseline Age | -0.041*** | -0.041*** | -0.021*** | -0.021*** | -0.018*** | -0.018*** |
|  | [-0.044,-0.038] | [-0.044,-0.038] | [-0.023,-0.018] | [-0.023,-0.018] | [-0.021,-0.014] | [-0.022,-0.014] |
| Race |  |  |  |  |  |  |
| NH-Black | -0.185*** | -0.184*** | -0.237*** | -0.238*** | -0.117*** | -0.130*** |
|  | [-0.237,-0.133] | [-0.236,-0.132] | [-0.288,-0.186] | [-0.289,-0.187] | [-0.172,-0.063] | [-0.184,-0.077] |
| NH-AAPI | -0.161* | -0.159* | -0.159 | -0.159 | -0.258** | -0.264*** |
|  | [-0.301,-0.021] | [-0.299,-0.018] | [-0.332,0.015] | [-0.332,0.014] | [-0.408,-0.109] | [-0.416,-0.113] |
| Hispanic | -0.199*** | -0.196*** | -0.278*** | -0.280*** | -0.360*** | -0.380*** |
|  | [-0.305,-0.093] | [-0.301,-0.090] | [-0.360,-0.197] | [-0.361,-0.199] | [-0.473,-0.247] | [-0.491,-0.269] |
| NA | -0.190*** | -0.181*** | -0.035 | -0.039 | 0.017 | -0.03 |
|  | [-0.234,-0.146] | [-0.228,-0.134] | [-0.077,0.007] | [-0.085,0.007] | [-0.022,0.056] | [-0.073,0.013] |
| College Degree or Beyond | 0.356*** | 0.356*** | 0.157*** | 0.158*** | 0.268*** | 0.270*** |
|  | [0.296,0.417] | [0.295,0.417] | [0.118,0.197] | [0.119,0.197] | [0.226,0.310] | [0.228,0.312] |
| Living with someone | 0.065*** | 0.065*** | 0.003 | 0.002 | 0.038 | 0.035 |
|  | [0.033,0.096] | [0.033,0.097] | [-0.036,0.041] | [-0.036,0.041] | [-0.005,0.080] | [-0.008,0.078] |
| Number of difficulties in ADL |  |  |  |  |  |  |
| 1 | -0.027 | -0.027 | -0.021 | -0.021 | -0.067*** | -0.067*** |
|  | [-0.061,0.006] | [-0.060,0.006] | [-0.061,0.019] | [-0.061,0.019] | [-0.103,-0.031] | [-0.103,-0.032] |
| 2 | -0.035 | -0.035 | -0.075*** | -0.075*** | -0.077** | -0.079** |
|  | [-0.077,0.007] | [-0.078,0.007] | [-0.118,-0.032] | [-0.118,-0.032] | [-0.130,-0.025] | [-0.132,-0.027] |
| 3 | -0.100** | -0.100** | -0.121** | -0.121** | -0.114*** | -0.113*** |
|  | [-0.168,-0.031] | [-0.168,-0.031] | [-0.197,-0.044] | [-0.197,-0.044] | [-0.171,-0.057] | [-0.171,-0.056] |
| 4 | -0.114** | -0.112** | -0.097* | -0.097* | -0.213*** | -0.211*** |
|  | [-0.183,-0.045] | [-0.181,-0.043] | [-0.189,-0.005] | [-0.189,-0.005] | [-0.310,-0.116] | [-0.306,-0.115] |
| 5 | -0.187*** | -0.185*** | -0.167** | -0.167** | -0.228*** | -0.227*** |
|  | [-0.261,-0.112] | [-0.260,-0.110] | [-0.277,-0.057] | [-0.277,-0.056] | [-0.331,-0.126] | [-0.328,-0.125] |
| 6 | -0.176* | -0.175* | -0.109 | -0.11 | -0.321*** | -0.324*** |
|  | [-0.314,-0.037] | [-0.314,-0.037] | [-0.236,0.017] | [-0.236,0.016] | [-0.428,-0.215] | [-0.428,-0.220] |
| 7 | -0.334** | -0.332** | -0.372** | -0.372** | -0.255* | -0.249* |
|  | [-0.529,-0.139] | [-0.525,-0.139] | [-0.640,-0.105] | [-0.640,-0.104] | [-0.452,-0.058] | [-0.443,-0.055] |
| NA | -0.285*** | -0.284*** | -0.370*** | -0.369*** | -0.484*** | -0.479*** |
|  | [-0.347,-0.222] | [-0.346,-0.222] | [-0.450,-0.289] | [-0.450,-0.289] | [-0.563,-0.405] | [-0.558,-0.400] |
| Number of difficulties in IADL |  |  |  |  |  |  |
| 1 | -0.028 | -0.028 | 0.012 | 0.012 | 0.019 | 0.02 |
|  | [-0.066,0.010] | [-0.066,0.010] | [-0.042,0.066] | [-0.042,0.066] | [-0.023,0.061] | [-0.022,0.063] |
| 2 | -0.002 | -0.002 | 0.025 | 0.025 | -0.002 | -0.001 |
|  | [-0.092,0.088] | [-0.092,0.088] | [-0.050,0.101] | [-0.050,0.101] | [-0.067,0.063] | [-0.067,0.064] |
| 3 | -0.116* | -0.118* | 0.039 | 0.038 | 0.037 | 0.026 |
|  | [-0.220,-0.012] | [-0.223,-0.014] | [-0.052,0.129] | [-0.052,0.128] | [-0.059,0.132] | [-0.070,0.123] |
| 4 | -0.058 | -0.06 | 0.031 | 0.031 | 0.001 | 0 |
|  | [-0.250,0.134] | [-0.251,0.131] | [-0.168,0.229] | [-0.168,0.229] | [-0.184,0.187] | [-0.188,0.188] |
| 5 | 0.131 | 0.126 | -0.051 | -0.052 | -0.105 | -0.111 |
|  | [-0.104,0.367] | [-0.111,0.362] | [-0.319,0.216] | [-0.319,0.215] | [-0.236,0.025] | [-0.238,0.017] |
| NA | -0.106*** | -0.106*** | -0.052** | -0.052** | -0.125*** | -0.124*** |
|  | [-0.140,-0.072] | [-0.140,-0.072] | [-0.082,-0.022] | [-0.082,-0.022] | [-0.154,-0.096] | [-0.153,-0.095] |
| Self-Rated Health |  |  |  |  |  |  |
| Very Good | -0.004 | -0.004 | 0.023 | 0.023 | 0.017 | 0.018 |
|  | [-0.035,0.027] | [-0.035,0.026] | [-0.025,0.072] | [-0.025,0.072] | [-0.026,0.061] | [-0.025,0.060] |
| Good | -0.032 | -0.032 | -0.006 | -0.006 | 0.007 | 0.005 |
|  | [-0.079,0.014] | [-0.078,0.014] | [-0.051,0.040] | [-0.051,0.040] | [-0.044,0.059] | [-0.046,0.057] |
| Fair | -0.099*** | -0.098*** | -0.068* | -0.068* | -0.02 | -0.022 |
|  | [-0.155,-0.043] | [-0.154,-0.043] | [-0.124,-0.012] | [-0.124,-0.012] | [-0.078,0.038] | [-0.079,0.036] |
| Poor | -0.192*** | -0.192*** | -0.167** | -0.167** | -0.022 | -0.025 |
|  | [-0.280,-0.104] | [-0.279,-0.104] | [-0.270,-0.063] | [-0.270,-0.064] | [-0.129,0.084] | [-0.131,0.081] |
| NA | -0.19 | -0.193 | 0.528*** | 0.528*** | 0.124 | 0.121 |
|  | [-1.002,0.622] | [-1.005,0.618] | [0.316,0.740] | [0.316,0.739] | [-0.202,0.449] | [-0.212,0.454] |
| Non-Metropolitan | -0.042 | -0.042 | -0.017 | -0.017 | -0.029 | -0.027 |
|  | [-0.099,0.015] | [-0.099,0.015] | [-0.070,0.036] | [-0.070,0.036] | [-0.091,0.034] | [-0.089,0.035] |
| Variance of Constant | 0.363*** | 0.363*** | 0.264*** | 0.264*** | 0.336*** | 0.335*** |
|  | [0.339,0.387] | [0.339,0.386] | [0.247,0.281] | [0.247,0.281] | [0.312,0.360] | [0.312,0.359] |
| Variance of Cognition | 0.396*** | 0.396*** | 0.563*** | 0.563*** | 0.399*** | 0.397*** |
|  | [0.383,0.409] | [0.382,0.409] | [0.539,0.586] | [0.539,0.586] | [0.381,0.417] | [0.380,0.415] |
| Observations | 31038 | 31038 | 31187 | 31187 | 31038 | 31038 |
| 95% CIs in brackets  **p*<0.05, ***p*<0.01, ****p*<0.001 | | | | | | |
| **Asymmetric Random Effects Model of Online Banking and Cognitive Domains (Z-Scores)** | | | | | | |
|  | Episodic Memory  (Model A) | Episodic Memory  (Model B) | Executive Function  (Model A) | Executive Function  (Model B) | Orientation (Model A) | Orientation (Model B) |
| Intercept | 2.945*** | 2.947*** | 1.354*** | 1.356*** | 1.077*** | 1.090*** |
|  | [2.649,3.240] | [2.652,3.242] | [1.087,1.621] | [1.088,1.624] | [0.751,1.403] | [0.764,1.416] |
| Transition in Banking | 0.049 | -0.085 | 0.061* | -0.108 | 0.077** | -0.326** |
|  | [-0.003,0.100] | [-0.214,0.044] | [0.010,0.112] | [-0.307,0.091] | [0.032,0.123] | [-0.516,-0.136] |
| Transition out Banking | -0.078** | -0.001 | -0.059* | 0.08 | -0.062 | -0.141 |
|  | [-0.129,-0.026] | [-0.180,0.179] | [-0.117,-0.000] | [-0.151,0.310] | [-0.126,0.003] | [-0.330,0.048] |
| Time | -0.026*** | -0.026*** | -0.006 | -0.006 | -0.012** | -0.012*** |
|  | [-0.032,-0.020] | [-0.033,-0.020] | [-0.013,0.002] | [-0.013,0.002] | [-0.018,-0.005] | [-0.019,-0.005] |
| Transition in Banking * Time |  | 0.016 |  | 0.02 |  | 0.044*** |
|  |  | [-0.001,0.032] |  | [-0.003,0.043] |  | [0.021,0.067] |
| Transition out Banking * Time |  | -0.01 |  | -0.017 |  | 0.005 |
|  |  | [-0.030,0.011] |  | [-0.045,0.010] |  | [-0.016,0.026] |
| Female | 0.298*** | 0.298*** | 0.046 | 0.046 | -0.015 | -0.015 |
|  | [0.255,0.340] | [0.255,0.340] | [-0.006,0.099] | [-0.006,0.099] | [-0.053,0.022] | [-0.053,0.022] |
| Number of Device Ownership (Cellphone, Computer, and Tablet) | | | | | | |
| 1 | 0.115** | 0.115** | 0.203*** | 0.203*** | 0.311*** | 0.312*** |
|  | [0.034,0.195] | [0.034,0.195] | [0.136,0.271] | [0.136,0.270] | [0.220,0.402] | [0.221,0.403] |
| 2 | 0.253*** | 0.253*** | 0.349*** | 0.349*** | 0.468*** | 0.472*** |
|  | [0.176,0.331] | [0.176,0.331] | [0.272,0.427] | [0.272,0.426] | [0.373,0.564] | [0.377,0.567] |
| 3 | 0.284*** | 0.284*** | 0.380*** | 0.380*** | 0.518*** | 0.523*** |
|  | [0.198,0.370] | [0.198,0.370] | [0.297,0.463] | [0.298,0.461] | [0.429,0.606] | [0.435,0.611] |
| Baseline Age | -0.041*** | -0.041*** | -0.021*** | -0.021*** | -0.018*** | -0.018*** |
|  | [-0.045,-0.038] | [-0.045,-0.038] | [-0.023,-0.018] | [-0.023,-0.018] | [-0.022,-0.014] | [-0.022,-0.014] |
| Race |  |  |  |  |  |  |
| NH-Black | -0.183*** | -0.184*** | -0.238*** | -0.238*** | -0.115*** | -0.123*** |
|  | [-0.235,-0.132] | [-0.236,-0.132] | [-0.289,-0.187] | [-0.290,-0.186] | [-0.170,-0.060] | [-0.177,-0.068] |
| NH-AAPI | -0.166* | -0.165* | -0.162 | -0.161 | -0.264*** | -0.257*** |
|  | [-0.306,-0.027] | [-0.303,-0.027] | [-0.336,0.011] | [-0.334,0.011] | [-0.414,-0.115] | [-0.396,-0.119] |
| Hispanic | -0.197*** | -0.199*** | -0.280*** | -0.281*** | -0.357*** | -0.365*** |
|  | [-0.305,-0.090] | [-0.306,-0.092] | [-0.361,-0.198] | [-0.362,-0.200] | [-0.472,-0.243] | [-0.479,-0.252] |
| NA | -0.189*** | -0.193*** | -0.035 | -0.038 | 0.017 | -0.011 |
|  | [-0.234,-0.145] | [-0.240,-0.147] | [-0.078,0.007] | [-0.081,0.006] | [-0.022,0.056] | [-0.051,0.028] |
| College Degree or Beyond | 0.365*** | 0.365*** | 0.163*** | 0.163*** | 0.280*** | 0.280*** |
|  | [0.305,0.426] | [0.305,0.426] | [0.123,0.202] | [0.124,0.202] | [0.238,0.322] | [0.238,0.322] |
| Living with someone | 0.066*** | 0.066*** | 0.003 | 0.003 | 0.039 | 0.04 |
|  | [0.035,0.097] | [0.035,0.097] | [-0.035,0.042] | [-0.036,0.042] | [-0.003,0.082] | [-0.002,0.083] |
| Number of difficulties in ADL |  |  |  |  |  |  |
| 1 | -0.028 | -0.028 | -0.021 | -0.021 | -0.067*** | -0.067*** |
|  | [-0.061,0.006] | [-0.061,0.005] | [-0.061,0.020] | [-0.061,0.019] | [-0.103,-0.032] | [-0.103,-0.032] |
| 2 | -0.034 | -0.034 | -0.074** | -0.074** | -0.077** | -0.077** |
|  | [-0.076,0.008] | [-0.075,0.008] | [-0.117,-0.031] | [-0.117,-0.031] | [-0.129,-0.024] | [-0.130,-0.024] |
| 3 | -0.100** | -0.100** | -0.120** | -0.120** | -0.113*** | -0.113*** |
|  | [-0.168,-0.031] | [-0.169,-0.031] | [-0.196,-0.043] | [-0.197,-0.043] | [-0.169,-0.057] | [-0.169,-0.056] |
| 4 | -0.114** | -0.113** | -0.096* | -0.095* | -0.213*** | -0.211*** |
|  | [-0.183,-0.044] | [-0.182,-0.043] | [-0.188,-0.004] | [-0.187,-0.004] | [-0.310,-0.116] | [-0.307,-0.115] |
| 5 | -0.187*** | -0.187*** | -0.167** | -0.166** | -0.230*** | -0.228*** |
|  | [-0.261,-0.114] | [-0.261,-0.113] | [-0.277,-0.057] | [-0.276,-0.057] | [-0.331,-0.129] | [-0.328,-0.127] |
| 6 | -0.175* | -0.174* | -0.108 | -0.107 | -0.320*** | -0.317*** |
|  | [-0.314,-0.036] | [-0.313,-0.035] | [-0.235,0.019] | [-0.234,0.019] | [-0.426,-0.213] | [-0.425,-0.210] |
| 7 | -0.339*** | -0.337** | -0.375** | -0.374** | -0.263** | -0.256* |
|  | [-0.533,-0.144] | [-0.532,-0.142] | [-0.642,-0.109] | [-0.641,-0.107] | [-0.457,-0.068] | [-0.450,-0.063] |
| NA | -0.287*** | -0.287*** | -0.371*** | -0.371*** | -0.490*** | -0.487*** |
|  | [-0.350,-0.225] | [-0.350,-0.224] | [-0.451,-0.291] | [-0.451,-0.291] | [-0.568,-0.411] | [-0.567,-0.408] |
| Number of difficulties in IADL |  |  |  |  |  |  |
| 1 | -0.029 | -0.029 | 0.011 | 0.011 | 0.018 | 0.017 |
|  | [-0.067,0.010] | [-0.067,0.010] | [-0.043,0.065] | [-0.043,0.065] | [-0.024,0.060] | [-0.025,0.059] |
| 2 | -0.001 | -0.001 | 0.025 | 0.025 | -0.003 | -0.005 |
|  | [-0.091,0.088] | [-0.091,0.088] | [-0.051,0.101] | [-0.050,0.101] | [-0.068,0.061] | [-0.070,0.060] |
| 3 | -0.116* | -0.116* | 0.039 | 0.038 | 0.037 | 0.035 |
|  | [-0.220,-0.012] | [-0.220,-0.013] | [-0.053,0.130] | [-0.053,0.130] | [-0.057,0.131] | [-0.056,0.127] |
| 4 | -0.055 | -0.056 | 0.032 | 0.031 | 0.004 | 0.005 |
|  | [-0.246,0.136] | [-0.246,0.135] | [-0.167,0.230] | [-0.168,0.230] | [-0.180,0.188] | [-0.178,0.188] |
| 5 | 0.132 | 0.13 | -0.052 | -0.055 | -0.105 | -0.11 |
|  | [-0.104,0.368] | [-0.106,0.366] | [-0.321,0.217] | [-0.324,0.214] | [-0.235,0.025] | [-0.237,0.018] |
| NA | -0.107*** | -0.106*** | -0.052*** | -0.052*** | -0.128*** | -0.127*** |
|  | [-0.141,-0.072] | [-0.140,-0.072] | [-0.082,-0.022] | [-0.082,-0.022] | [-0.156,-0.100] | [-0.156,-0.099] |
| Self-Rated Health |  |  |  |  |  |  |
| Very Good | -0.004 | -0.004 | 0.023 | 0.023 | 0.018 | 0.019 |
|  | [-0.034,0.027] | [-0.034,0.027] | [-0.025,0.071] | [-0.025,0.071] | [-0.025,0.061] | [-0.024,0.061] |
| Good | -0.031 | -0.031 | -0.005 | -0.005 | 0.008 | 0.009 |
|  | [-0.077,0.016] | [-0.077,0.016] | [-0.050,0.040] | [-0.050,0.040] | [-0.043,0.060] | [-0.042,0.060] |
| Fair | -0.099*** | -0.098*** | -0.068* | -0.068* | -0.02 | -0.018 |
|  | [-0.155,-0.042] | [-0.155,-0.042] | [-0.124,-0.012] | [-0.124,-0.012] | [-0.077,0.038] | [-0.075,0.038] |
| Poor | -0.192*** | -0.192*** | -0.167** | -0.167** | -0.023 | -0.022 |
|  | [-0.281,-0.104] | [-0.281,-0.104] | [-0.270,-0.065] | [-0.270,-0.064] | [-0.130,0.083] | [-0.128,0.084] |
| NA | -0.198 | -0.199 | 0.523*** | 0.521*** | 0.116 | 0.109 |
|  | [-1.010,0.615] | [-1.011,0.613] | [0.309,0.736] | [0.307,0.735] | [-0.210,0.441] | [-0.217,0.436] |
| Non-Metropolitan | -0.044 | -0.044 | -0.019 | -0.018 | -0.032 | -0.03 |
|  | [-0.102,0.013] | [-0.101,0.014] | [-0.072,0.035] | [-0.071,0.035] | [-0.095,0.030] | [-0.093,0.032] |
| Variance of Constant | 0.364*** | 0.364*** | 0.264*** | 0.264*** | 0.337*** | 0.336*** |
|  | [0.340,0.388] | [0.340,0.388] | [0.247,0.281] | [0.247,0.280] | [0.313,0.361] | [0.313,0.360] |
| Variance of Cognition | 0.396*** | 0.396*** | 0.563*** | 0.562*** | 0.400*** | 0.399*** |
|  | [0.383,0.409] | [0.383,0.409] | [0.539,0.586] | [0.539,0.586] | [0.382,0.418] | [0.381,0.417] |
| Observations | 31038 | 31038 | 31187 | 31187 | 31038 | 31038 |
| 95% confidence intervals in brackets  * *p*<0.05, ** *p*<0.01, *** *p*<0.001 | | | | | | |

| **Appendix B3. Asymmetric Random Effects Model of Online Medication Refills and Cognitive Domains (Z-Scores)** | | | | | | |
| --- | --- | --- | --- | --- | --- | --- |
|  | Episodic Memory  (Model A) | Episodic Memory  (Model B) | Executive Function  (Model A) | Executive Function  (Model B) | Orientation (Model A) | Orientation (Model B) |
| Intercept | 2.963*** | 2.954*** | 1.365*** | 1.370*** | 1.096*** | 1.149*** |
|  | [2.670,3.257] | [2.661,3.246] | [1.100,1.631] | [1.103,1.638] | [0.768,1.424] | [0.824,1.473] |
| Transition in Med Refills | 0.073*** | 0.09 | 0.026 | -0.102 | 0.097*** | -0.372*** |
|  | [0.038,0.109] | [-0.042,0.222] | [-0.014,0.067] | [-0.300,0.096] | [0.054,0.140] | [-0.516,-0.228] |
| Transition out Med Refills | -0.014 | 0.101 | 0.011 | 0.117 | -0.007 | -0.104 |
|  | [-0.068,0.040] | [-0.109,0.311] | [-0.038,0.059] | [-0.142,0.376] | [-0.055,0.040] | [-0.241,0.033] |
| Time | -0.032*** | -0.032*** | -0.008* | -0.008* | -0.018*** | -0.019*** |
|  | [-0.039,-0.025] | [-0.039,-0.025] | [-0.015,-0.001] | [-0.015,-0.001] | [-0.025,-0.011] | [-0.026,-0.012] |
| Transition in Med Refills * Time |  | -0.001 |  | 0.015 |  | 0.052*** |
|  |  | [-0.017,0.015] |  | [-0.006,0.037] |  | [0.036,0.068] |
| Transition out Med Refills * Time |  | -0.012 |  | -0.013 |  | 0.005 |
|  |  | [-0.037,0.012] |  | [-0.042,0.016] |  | [-0.012,0.021] |
| Female | 0.299*** | 0.300*** | 0.047 | 0.047 | -0.014 | -0.016 |
|  | [0.256,0.342] | [0.257,0.343] | [-0.006,0.100] | [-0.005,0.100] | [-0.052,0.024] | [-0.053,0.021] |
| Number of Device Ownership (Cellphone, Computer, and Tablet) | | | | | | |
| 1 | 0.113** | 0.113** | 0.203*** | 0.203*** | 0.309*** | 0.310*** |
|  | [0.032,0.193] | [0.032,0.193] | [0.135,0.270] | [0.135,0.270] | [0.219,0.400] | [0.219,0.400] |
| 2 | 0.247*** | 0.246*** | 0.347*** | 0.347*** | 0.462*** | 0.465*** |
|  | [0.169,0.325] | [0.168,0.324] | [0.269,0.424] | [0.269,0.424] | [0.367,0.557] | [0.371,0.559] |
| 3 | 0.278*** | 0.276*** | 0.377*** | 0.377*** | 0.510*** | 0.515*** |
|  | [0.191,0.364] | [0.189,0.363] | [0.294,0.460] | [0.294,0.460] | [0.421,0.598] | [0.427,0.603] |
| Baseline Age | -0.041*** | -0.041*** | -0.021*** | -0.021*** | -0.018*** | -0.018*** |
|  | [-0.044,-0.038] | [-0.044,-0.037] | [-0.023,-0.018] | [-0.023,-0.018] | [-0.022,-0.014] | [-0.022,-0.014] |
| Race |  |  |  |  |  |  |
| NH-Black | -0.185*** | -0.183*** | -0.237*** | -0.238*** | -0.117*** | -0.128*** |
|  | [-0.236,-0.133] | [-0.234,-0.131] | [-0.288,-0.186] | [-0.289,-0.187] | [-0.172,-0.062] | [-0.183,-0.074] |
| NH-AAPI | -0.163* | -0.162* | -0.161 | -0.16 | -0.262*** | -0.261*** |
|  | [-0.302,-0.024] | [-0.302,-0.022] | [-0.333,0.012] | [-0.333,0.013] | [-0.409,-0.115] | [-0.402,-0.119] |
| Hispanic | -0.199*** | -0.195*** | -0.279*** | -0.280*** | -0.360*** | -0.377*** |
|  | [-0.306,-0.092] | [-0.302,-0.088] | [-0.361,-0.197] | [-0.361,-0.199] | [-0.474,-0.246] | [-0.490,-0.263] |
| NA | -0.191*** | -0.181*** | -0.036 | -0.04 | 0.015 | -0.036 |
|  | [-0.235,-0.147] | [-0.226,-0.135] | [-0.078,0.006] | [-0.082,0.002] | [-0.025,0.054] | [-0.077,0.005] |
| College Degree or Beyond | 0.359*** | 0.358*** | 0.159*** | 0.159*** | 0.272*** | 0.276*** |
|  | [0.298,0.419] | [0.297,0.418] | [0.120,0.199] | [0.120,0.199] | [0.230,0.313] | [0.234,0.318] |
| Living with someone | 0.064*** | 0.064*** | 0.003 | 0.002 | 0.037 | 0.037 |
|  | [0.033,0.096] | [0.033,0.095] | [-0.036,0.041] | [-0.036,0.041] | [-0.005,0.080] | [-0.007,0.080] |
| Number of difficulties in ADL |  |  |  |  |  |  |
| 1 | -0.028 | -0.028 | -0.021 | -0.021 | -0.068*** | -0.067*** |
|  | [-0.061,0.005] | [-0.060,0.005] | [-0.061,0.019] | [-0.061,0.019] | [-0.103,-0.032] | [-0.103,-0.032] |
| 2 | -0.036 | -0.035 | -0.076*** | -0.076*** | -0.078** | -0.079** |
|  | [-0.078,0.007] | [-0.078,0.008] | [-0.119,-0.033] | [-0.119,-0.033] | [-0.131,-0.026] | [-0.131,-0.027] |
| 3 | -0.099** | -0.099** | -0.120** | -0.120** | -0.113*** | -0.112*** |
|  | [-0.168,-0.030] | [-0.168,-0.030] | [-0.197,-0.044] | [-0.196,-0.043] | [-0.169,-0.057] | [-0.168,-0.055] |
| 4 | -0.115** | -0.115** | -0.098* | -0.098* | -0.215*** | -0.214*** |
|  | [-0.184,-0.046] | [-0.184,-0.046] | [-0.190,-0.006] | [-0.190,-0.006] | [-0.312,-0.118] | [-0.310,-0.117] |
| 5 | -0.186*** | -0.185*** | -0.167** | -0.168** | -0.228*** | -0.234*** |
|  | [-0.260,-0.112] | [-0.260,-0.111] | [-0.277,-0.057] | [-0.279,-0.058] | [-0.330,-0.127] | [-0.336,-0.132] |
| 6 | -0.174* | -0.174* | -0.109 | -0.109 | -0.319*** | -0.318*** |
|  | [-0.314,-0.035] | [-0.314,-0.034] | [-0.236,0.017] | [-0.235,0.018] | [-0.425,-0.214] | [-0.421,-0.215] |
| 7 | -0.341*** | -0.340*** | -0.376** | -0.377** | -0.265** | -0.268** |
|  | [-0.535,-0.146] | [-0.534,-0.146] | [-0.641,-0.112] | [-0.641,-0.113] | [-0.456,-0.073] | [-0.461,-0.075] |
| NA | -0.284*** | -0.285*** | -0.371*** | -0.371*** | -0.485*** | -0.483*** |
|  | [-0.347,-0.222] | [-0.347,-0.222] | [-0.451,-0.291] | [-0.451,-0.291] | [-0.565,-0.406] | [-0.563,-0.403] |
| Number of difficulties in IADL |  |  |  |  |  |  |
| 1 | -0.027 | -0.027 | 0.012 | 0.012 | 0.019 | 0.019 |
|  | [-0.066,0.011] | [-0.066,0.011] | [-0.042,0.066] | [-0.042,0.066] | [-0.022,0.061] | [-0.023,0.061] |
| 2 | -0.003 | -0.003 | 0.025 | 0.025 | -0.003 | -0.001 |
|  | [-0.092,0.087] | [-0.093,0.087] | [-0.051,0.100] | [-0.051,0.100] | [-0.069,0.063] | [-0.067,0.065] |
| 3 | -0.118* | -0.117* | 0.037 | 0.037 | 0.033 | 0.029 |
|  | [-0.222,-0.014] | [-0.221,-0.013] | [-0.053,0.128] | [-0.052,0.127] | [-0.063,0.130] | [-0.066,0.124] |
| 4 | -0.06 | -0.061 | 0.03 | 0.029 | -0.002 | -0.001 |
|  | [-0.254,0.133] | [-0.254,0.131] | [-0.169,0.228] | [-0.171,0.228] | [-0.187,0.183] | [-0.195,0.193] |
| 5 | 0.124 | 0.124 | -0.053 | -0.056 | -0.114 | -0.124 |
|  | [-0.117,0.364] | [-0.117,0.364] | [-0.321,0.216] | [-0.324,0.211] | [-0.245,0.016] | [-0.255,0.007] |
| NA | -0.106*** | -0.106*** | -0.053*** | -0.052*** | -0.127*** | -0.127*** |
|  | [-0.141,-0.071] | [-0.141,-0.071] | [-0.083,-0.022] | [-0.083,-0.022] | [-0.155,-0.098] | [-0.155,-0.099] |
| Self-Rated Health |  |  |  |  |  |  |
| Very Good | -0.004 | -0.004 | 0.023 | 0.023 | 0.017 | 0.015 |
|  | [-0.035,0.027] | [-0.035,0.027] | [-0.025,0.071] | [-0.026,0.071] | [-0.026,0.060] | [-0.027,0.057] |
| Good | -0.032 | -0.032 | -0.006 | -0.006 | 0.007 | 0.005 |
|  | [-0.078,0.014] | [-0.078,0.015] | [-0.051,0.040] | [-0.052,0.039] | [-0.044,0.059] | [-0.045,0.056] |
| Fair | -0.099*** | -0.099*** | -0.068* | -0.069* | -0.02 | -0.023 |
|  | [-0.155,-0.044] | [-0.154,-0.043] | [-0.124,-0.012] | [-0.125,-0.013] | [-0.079,0.038] | [-0.080,0.035] |
| Poor | -0.193*** | -0.193*** | -0.167** | -0.167** | -0.024 | -0.02 |
|  | [-0.281,-0.104] | [-0.282,-0.105] | [-0.270,-0.065] | [-0.270,-0.064] | [-0.130,0.082] | [-0.126,0.085] |
| NA | -0.182 | -0.182 | 0.534*** | 0.529*** | 0.137 | 0.122 |
|  | [-0.984,0.620] | [-0.978,0.614] | [0.315,0.752] | [0.311,0.748] | [-0.176,0.451] | [-0.199,0.442] |
| Non-Metropolitan | -0.041 | -0.04 | -0.017 | -0.017 | -0.027 | -0.028 |
|  | [-0.099,0.017] | [-0.098,0.018] | [-0.070,0.036] | [-0.070,0.037] | [-0.090,0.035] | [-0.089,0.033] |
| Variance of Constant | 0.365*** | 0.365*** | 0.264*** | 0.264*** | 0.337*** | 0.336*** |
|  | [0.340,0.389] | [0.340,0.389] | [0.248,0.281] | [0.247,0.281] | [0.313,0.361] | [0.312,0.360] |
| Variance of Cognition | 0.396*** | 0.396*** | 0.563*** | 0.562*** | 0.399*** | 0.397*** |
|  | [0.383,0.409] | [0.382,0.409] | [0.539,0.586] | [0.539,0.586] | [0.381,0.417] | [0.379,0.415] |
| Observations | 31038 | 31038 | 31187 | 31187 | 31038 | 31038 |
| 95% CIs in brackets  **p*<0.05, ***p*<0.01, ****p*<0.001 | | | | | | |
| **Asymmetric Random Effects Model of Visiting Social Network Sites Online and Cognitive Domains (Z-Scores)** | | | | | | |
|  | Episodic Memory  (Model A) | Episodic Memory  (Model B) | Executive Function  (Model A) | Executive Function  (Model B) | Orientation (Model A) | Orientation (Model B) |
| Intercept | 2.951*** | 2.946*** | 1.358*** | 1.357*** | 1.088*** | 1.112*** |
|  | [2.657,3.245] | [2.649,3.242] | [1.090,1.626] | [1.091,1.623] | [0.760,1.416] | [0.787,1.438] |
| Transition in Social Media | 0.065** | -0.012 | 0.05 | 0.119 | 0.080*** | -0.268*** |
|  | [0.022,0.109] | [-0.139,0.115] | [-0.002,0.101] | [-0.024,0.262] | [0.041,0.119] | [-0.386,-0.151] |
| Transition out Social Media | -0.066** | 0.124 | -0.045 | -0.109 | -0.024 | -0.043 |
|  | [-0.105,-0.028] | [-0.031,0.278] | [-0.094,0.004] | [-0.286,0.069] | [-0.068,0.021] | [-0.179,0.094] |
| Time | -0.027*** | -0.027*** | -0.006 | -0.006 | -0.015*** | -0.015*** |
|  | [-0.034,-0.020] | [-0.034,-0.020] | [-0.013,0.001] | [-0.012,0.001] | [-0.022,-0.007] | [-0.023,-0.008] |
| Transition in Social Media * Time |  | 0.01 |  | -0.008 |  | 0.039*** |
|  |  | [-0.005,0.026] |  | [-0.026,0.009] |  | [0.025,0.053] |
| Transition out Social Media * Time |  | -0.022* |  | 0.008 |  | -0.002 |
|  |  | [-0.040,-0.004] |  | [-0.014,0.030] |  | [-0.018,0.015] |
| Female | 0.299*** | 0.299*** | 0.047 | 0.047 | -0.013 | -0.014 |
|  | [0.257,0.342] | [0.257,0.342] | [-0.005,0.100] | [-0.005,0.100] | [-0.051,0.024] | [-0.052,0.023] |
| Number of Device Ownership (Cellphone, Computer, and Tablet) | | | | | | |
| 1 | 0.114** | 0.113** | 0.203*** | 0.204*** | 0.309*** | 0.310*** |
|  | [0.034,0.195] | [0.033,0.194] | [0.136,0.271] | [0.136,0.271] | [0.218,0.400] | [0.219,0.401] |
| 2 | 0.250*** | 0.249*** | 0.348*** | 0.348*** | 0.464*** | 0.469*** |
|  | [0.172,0.328] | [0.171,0.326] | [0.270,0.427] | [0.269,0.427] | [0.368,0.559] | [0.373,0.564] |
| 3 | 0.282*** | 0.280*** | 0.380*** | 0.379*** | 0.513*** | 0.520*** |
|  | [0.195,0.368] | [0.194,0.366] | [0.295,0.464] | [0.295,0.464] | [0.425,0.602] | [0.431,0.609] |
| Baseline Age | -0.041*** | -0.041*** | -0.021*** | -0.021*** | -0.018*** | -0.018*** |
|  | [-0.045,-0.038] | [-0.045,-0.038] | [-0.023,-0.018] | [-0.023,-0.018] | [-0.022,-0.014] | [-0.022,-0.014] |
| Race |  |  |  |  |  |  |
| NH-Black | -0.182*** | -0.181*** | -0.237*** | -0.237*** | -0.115*** | -0.119*** |
|  | [-0.234,-0.131] | [-0.233,-0.129] | [-0.288,-0.186] | [-0.288,-0.186] | [-0.170,-0.059] | [-0.174,-0.063] |
| NH-AAPI | -0.165* | -0.165* | -0.162 | -0.162 | -0.265*** | -0.263*** |
|  | [-0.305,-0.026] | [-0.304,-0.026] | [-0.336,0.012] | [-0.336,0.011] | [-0.414,-0.115] | [-0.410,-0.116] |
| Hispanic | -0.194*** | -0.193*** | -0.277*** | -0.277*** | -0.354*** | -0.359*** |
|  | [-0.301,-0.086] | [-0.301,-0.086] | [-0.359,-0.195] | [-0.359,-0.195] | [-0.468,-0.239] | [-0.474,-0.244] |
| NA | -0.188*** | -0.179*** | -0.034 | -0.034 | 0.017 | -0.013 |
|  | [-0.232,-0.144] | [-0.227,-0.131] | [-0.077,0.008] | [-0.079,0.011] | [-0.022,0.056] | [-0.053,0.027] |
| College Degree or Beyond | 0.366*** | 0.366*** | 0.163*** | 0.164*** | 0.280*** | 0.279*** |
|  | [0.305,0.427] | [0.305,0.427] | [0.124,0.203] | [0.124,0.203] | [0.238,0.322] | [0.237,0.321] |
| Living with someone | 0.065*** | 0.065*** | 0.003 | 0.003 | 0.039 | 0.038 |
|  | [0.034,0.096] | [0.035,0.096] | [-0.036,0.042] | [-0.035,0.042] | [-0.003,0.082] | [-0.004,0.081] |
| Number of difficulties in ADL |  |  |  |  |  |  |
| 1 | -0.028 | -0.028 | -0.021 | -0.021 | -0.068*** | -0.068*** |
|  | [-0.061,0.005] | [-0.061,0.005] | [-0.062,0.019] | [-0.061,0.019] | [-0.104,-0.033] | [-0.103,-0.032] |
| 2 | -0.036 | -0.036 | -0.075*** | -0.075*** | -0.079** | -0.079** |
|  | [-0.078,0.006] | [-0.077,0.006] | [-0.119,-0.032] | [-0.119,-0.032] | [-0.131,-0.027] | [-0.130,-0.027] |
| 3 | -0.100** | -0.100** | -0.121** | -0.121** | -0.114*** | -0.113*** |
|  | [-0.168,-0.032] | [-0.168,-0.032] | [-0.197,-0.045] | [-0.197,-0.045] | [-0.170,-0.059] | [-0.169,-0.057] |
| 4 | -0.114** | -0.113** | -0.097* | -0.097* | -0.213*** | -0.213*** |
|  | [-0.183,-0.044] | [-0.183,-0.044] | [-0.189,-0.004] | [-0.189,-0.005] | [-0.309,-0.116] | [-0.310,-0.117] |
| 5 | -0.188*** | -0.187*** | -0.168** | -0.168** | -0.231*** | -0.232*** |
|  | [-0.262,-0.114] | [-0.260,-0.113] | [-0.277,-0.059] | [-0.278,-0.059] | [-0.333,-0.129] | [-0.333,-0.131] |
| 6 | -0.174* | -0.174* | -0.108 | -0.108 | -0.320*** | -0.322*** |
|  | [-0.314,-0.035] | [-0.313,-0.035] | [-0.235,0.018] | [-0.235,0.018] | [-0.425,-0.214] | [-0.426,-0.219] |
| 7 | -0.340*** | -0.340*** | -0.376** | -0.376** | -0.263** | -0.259* |
|  | [-0.534,-0.145] | [-0.535,-0.146] | [-0.643,-0.109] | [-0.644,-0.109] | [-0.459,-0.067] | [-0.459,-0.060] |
| NA | -0.288*** | -0.288*** | -0.372*** | -0.373*** | -0.490*** | -0.486*** |
|  | [-0.351,-0.225] | [-0.352,-0.225] | [-0.453,-0.292] | [-0.453,-0.292] | [-0.567,-0.412] | [-0.563,-0.409] |
| Number of difficulties in IADL |  |  |  |  |  |  |
| 1 | -0.028 | -0.029 | 0.011 | 0.012 | 0.019 | 0.019 |
|  | [-0.067,0.010] | [-0.067,0.009] | [-0.043,0.066] | [-0.043,0.066] | [-0.023,0.060] | [-0.023,0.061] |
| 2 | -0.003 | -0.002 | 0.025 | 0.024 | -0.004 | -0.004 |
|  | [-0.093,0.086] | [-0.091,0.087] | [-0.051,0.100] | [-0.051,0.100] | [-0.068,0.060] | [-0.067,0.059] |
| 3 | -0.114* | -0.114* | 0.04 | 0.04 | 0.04 | 0.037 |
|  | [-0.217,-0.011] | [-0.217,-0.011] | [-0.052,0.131] | [-0.052,0.131] | [-0.054,0.133] | [-0.055,0.130] |
| 4 | -0.052 | -0.049 | 0.035 | 0.034 | 0.009 | 0.012 |
|  | [-0.242,0.138] | [-0.238,0.139] | [-0.163,0.233] | [-0.164,0.232] | [-0.175,0.193] | [-0.171,0.195] |
| 5 | 0.133 | 0.136 | -0.05 | -0.051 | -0.099 | -0.099 |
|  | [-0.102,0.367] | [-0.099,0.370] | [-0.320,0.220] | [-0.321,0.219] | [-0.230,0.032] | [-0.228,0.030] |
| NA | -0.107*** | -0.106*** | -0.053*** | -0.053*** | -0.128*** | -0.128*** |
|  | [-0.141,-0.073] | [-0.140,-0.073] | [-0.083,-0.023] | [-0.084,-0.023] | [-0.156,-0.100] | [-0.156,-0.100] |
| Self-Rated Health |  |  |  |  |  |  |
| Very Good | -0.003 | -0.004 | 0.024 | 0.024 | 0.018 | 0.019 |
|  | [-0.034,0.028] | [-0.035,0.027] | [-0.025,0.072] | [-0.025,0.073] | [-0.025,0.062] | [-0.024,0.062] |
| Good | -0.03 | -0.031 | -0.005 | -0.005 | 0.008 | 0.009 |
|  | [-0.077,0.016] | [-0.077,0.015] | [-0.050,0.041] | [-0.050,0.041] | [-0.043,0.060] | [-0.043,0.060] |
| Fair | -0.098*** | -0.098*** | -0.067* | -0.067* | -0.019 | -0.019 |
|  | [-0.153,-0.042] | [-0.154,-0.043] | [-0.124,-0.011] | [-0.123,-0.011] | [-0.077,0.039] | [-0.076,0.039] |
| Poor | -0.191*** | -0.191*** | -0.166** | -0.166** | -0.023 | -0.02 |
|  | [-0.279,-0.102] | [-0.280,-0.102] | [-0.269,-0.063] | [-0.269,-0.063] | [-0.129,0.083] | [-0.127,0.086] |
| NA | -0.195 | -0.196 | 0.526*** | 0.526*** | 0.122 | 0.118 |
|  | [-1.014,0.623] | [-1.016,0.624] | [0.316,0.736] | [0.316,0.737] | [-0.202,0.446] | [-0.207,0.444] |
| Non-Metropolitan | -0.044 | -0.044 | -0.019 | -0.018 | -0.032 | -0.032 |
|  | [-0.102,0.013] | [-0.102,0.013] | [-0.071,0.034] | [-0.071,0.034] | [-0.094,0.030] | [-0.094,0.030] |
| Variance of Constant | 0.365*** | 0.365*** | 0.264*** | 0.264*** | 0.337*** | 0.337*** |
|  | [0.341,0.389] | [0.341,0.389] | [0.247,0.281] | [0.247,0.281] | [0.314,0.361] | [0.313,0.361] |
| Variance of Cognition | 0.396*** | 0.396*** | 0.563*** | 0.562*** | 0.400*** | 0.399*** |
|  | [0.382,0.409] | [0.382,0.409] | [0.539,0.586] | [0.539,0.586] | [0.382,0.418] | [0.381,0.417] |
| Observations | 31038 | 31038 | 31187 | 31187 | 31038 | 31038 |
| 95% CIs in brackets  **p*<0.05, ***p*<0.01, ****p*<0.001 | | | | | | |

| **Asymmetric Random Effects Model of Checking Health Information Online and Cognitive Domains (Z-Scores)** | | | | | | |
| --- | --- | --- | --- | --- | --- | --- |
|  | Episodic Memory  (Model A) | Episodic Memory  (Model B) | Executive Function  (Model A) | Executive Function  (Model B) | Orientation (Model A) | Orientation (Model B) |
| Intercept | 2.967*** | 2.961*** | 1.375*** | 1.382*** | 1.108*** | 1.173*** |
|  | [2.672,3.262] | [2.668,3.254] | [1.110,1.640] | [1.115,1.648] | [0.785,1.431] | [0.851,1.496] |
| Transition in Health Info Online | 0.047* | 0.047 | -0.011 | -0.048 | 0.098*** | -0.236*** |
|  | [0.005,0.089] | [-0.083,0.176] | [-0.054,0.031] | [-0.197,0.101] | [0.068,0.127] | [-0.361,-0.111] |
| Transition out Health Info Online | 0.027 | 0.081 | 0.060*** | 0.038 | 0.048* | -0.172** |
|  | [-0.014,0.067] | [-0.043,0.206] | [0.032,0.088] | [-0.128,0.204] | [0.010,0.085] | [-0.276,-0.068] |
| Time | -0.034*** | -0.034*** | -0.010** | -0.010** | -0.024*** | -0.026*** |
|  | [-0.042,-0.026] | [-0.042,-0.026] | [-0.016,-0.003] | [-0.017,-0.003] | [-0.031,-0.017] | [-0.032,-0.019] |
| Transition in Health Info Online * Time | | 0.001 |  | 0.004 |  | 0.036*** |
|  |  | [-0.014,0.015] |  | [-0.014,0.022] |  | [0.022,0.051] |
| Transition out Health Info Online * Time | | -0.006 |  | 0.002 |  | 0.020** |
|  |  | [-0.021,0.009] |  | [-0.018,0.022] |  | [0.005,0.034] |
| Female | 0.298*** | 0.298*** | 0.046 | 0.046 | -0.016 | -0.015 |
|  | [0.256,0.340] | [0.256,0.340] | [-0.006,0.099] | [-0.006,0.099] | [-0.053,0.021] | [-0.051,0.022] |
| Number of Device Ownership (Cellphone, Computer, and Tablet) | | | | | | |
| 1 | 0.112** | 0.112** | 0.202*** | 0.202*** | 0.307*** | 0.308*** |
|  | [0.031,0.193] | [0.031,0.193] | [0.135,0.269] | [0.135,0.269] | [0.217,0.398] | [0.218,0.398] |
| 2 | 0.245*** | 0.244*** | 0.344*** | 0.344*** | 0.454*** | 0.459*** |
|  | [0.166,0.323] | [0.165,0.323] | [0.266,0.421] | [0.267,0.421] | [0.359,0.549] | [0.365,0.553] |
| 3 | 0.276*** | 0.275*** | 0.375*** | 0.376*** | 0.501*** | 0.511*** |
|  | [0.188,0.363] | [0.187,0.362] | [0.293,0.457] | [0.294,0.457] | [0.414,0.589] | [0.424,0.599] |
| Baseline Age | -0.041*** | -0.041*** | -0.021*** | -0.021*** | -0.017*** | -0.018*** |
|  | [-0.044,-0.038] | [-0.044,-0.038] | [-0.023,-0.018] | [-0.023,-0.018] | [-0.021,-0.014] | [-0.022,-0.014] |
| Race |  |  |  |  |  |  |
| NH-Black | -0.185*** | -0.183*** | -0.238*** | -0.240*** | -0.118*** | -0.137*** |
|  | [-0.237,-0.133] | [-0.235,-0.130] | [-0.289,-0.187] | [-0.292,-0.188] | [-0.172,-0.063] | [-0.191,-0.082] |
| NH-AAPI | -0.163* | -0.163* | -0.163 | -0.163 | -0.260** | -0.258*** |
|  | [-0.303,-0.023] | [-0.304,-0.021] | [-0.337,0.010] | [-0.336,0.010] | [-0.411,-0.110] | [-0.397,-0.119] |
| Hispanic | -0.199*** | -0.196*** | -0.281*** | -0.283*** | -0.362*** | -0.389*** |
|  | [-0.306,-0.093] | [-0.301,-0.091] | [-0.362,-0.199] | [-0.366,-0.201] | [-0.474,-0.249] | [-0.502,-0.277] |
| NA | -0.193*** | -0.185*** | -0.045* | -0.052* | 0.011 | -0.050* |
|  | [-0.238,-0.148] | [-0.236,-0.134] | [-0.088,-0.003] | [-0.094,-0.009] | [-0.029,0.052] | [-0.096,-0.004] |
| College Degree or Beyond | 0.355*** | 0.355*** | 0.156*** | 0.156*** | 0.262*** | 0.267*** |
|  | [0.295,0.415] | [0.295,0.414] | [0.116,0.196] | [0.117,0.196] | [0.219,0.304] | [0.225,0.309] |
| Living with someone | 0.064*** | 0.064*** | 0.003 | 0.003 | 0.037 | 0.037 |
|  | [0.033,0.096] | [0.033,0.095] | [-0.035,0.042] | [-0.035,0.042] | [-0.005,0.079] | [-0.006,0.080] |
| Number of difficulties in ADL |  |  |  |  |  |  |
| 1 | -0.027 | -0.027 | -0.02 | -0.02 | -0.065*** | -0.066*** |
|  | [-0.060,0.007] | [-0.060,0.007] | [-0.060,0.020] | [-0.060,0.020] | [-0.101,-0.029] | [-0.102,-0.031] |
| 2 | -0.035 | -0.035 | -0.074*** | -0.074*** | -0.077** | -0.076** |
|  | [-0.078,0.008] | [-0.078,0.008] | [-0.117,-0.031] | [-0.117,-0.031] | [-0.129,-0.024] | [-0.128,-0.024] |
| 3 | -0.098** | -0.098** | -0.117** | -0.117** | -0.110*** | -0.110*** |
|  | [-0.167,-0.029] | [-0.167,-0.029] | [-0.194,-0.041] | [-0.194,-0.041] | [-0.166,-0.054] | [-0.166,-0.055] |
| 4 | -0.112** | -0.112** | -0.095* | -0.095* | -0.209*** | -0.210*** |
|  | [-0.182,-0.043] | [-0.182,-0.042] | [-0.187,-0.003] | [-0.187,-0.004] | [-0.307,-0.112] | [-0.307,-0.112] |
| 5 | -0.186*** | -0.186*** | -0.166** | -0.167** | -0.228*** | -0.231*** |
|  | [-0.261,-0.112] | [-0.260,-0.112] | [-0.276,-0.057] | [-0.276,-0.057] | [-0.330,-0.125] | [-0.335,-0.128] |
| 6 | -0.174* | -0.174* | -0.108 | -0.108 | -0.317*** | -0.317*** |
|  | [-0.314,-0.034] | [-0.315,-0.034] | [-0.234,0.018] | [-0.234,0.019] | [-0.425,-0.210] | [-0.422,-0.211] |
| 7 | -0.333** | -0.333** | -0.370** | -0.369** | -0.251* | -0.246* |
|  | [-0.528,-0.138] | [-0.528,-0.139] | [-0.638,-0.102] | [-0.637,-0.101] | [-0.448,-0.055] | [-0.448,-0.045] |
| NA | -0.283*** | -0.283*** | -0.370*** | -0.370*** | -0.480*** | -0.476*** |
|  | [-0.345,-0.221] | [-0.345,-0.221] | [-0.451,-0.289] | [-0.450,-0.289] | [-0.558,-0.401] | [-0.555,-0.398] |
| Number of difficulties in IADL |  |  |  |  |  |  |
| 1 | -0.028 | -0.028 | 0.012 | 0.012 | 0.018 | 0.019 |
|  | [-0.067,0.011] | [-0.067,0.011] | [-0.043,0.066] | [-0.043,0.066] | [-0.023,0.060] | [-0.021,0.060] |
| 2 | -0.002 | -0.002 | 0.026 | 0.026 | -0.001 | -0.003 |
|  | [-0.092,0.088] | [-0.091,0.088] | [-0.050,0.102] | [-0.050,0.102] | [-0.067,0.064] | [-0.069,0.063] |
| 3 | -0.113* | -0.112* | 0.041 | 0.041 | 0.043 | 0.045 |
|  | [-0.218,-0.008] | [-0.218,-0.007] | [-0.049,0.131] | [-0.049,0.131] | [-0.052,0.137] | [-0.047,0.136] |
| 4 | -0.057 | -0.056 | 0.03 | 0.03 | 0.003 | -0.001 |
|  | [-0.249,0.135] | [-0.248,0.135] | [-0.169,0.229] | [-0.169,0.228] | [-0.181,0.186] | [-0.187,0.185] |
| 5 | 0.138 | 0.137 | -0.045 | -0.044 | -0.092 | -0.086 |
|  | [-0.097,0.374] | [-0.099,0.373] | [-0.313,0.223] | [-0.312,0.223] | [-0.220,0.037] | [-0.215,0.043] |
| NA | -0.105*** | -0.105*** | -0.052** | -0.052** | -0.122*** | -0.120*** |
|  | [-0.140,-0.069] | [-0.140,-0.070] | [-0.082,-0.021] | [-0.082,-0.021] | [-0.151,-0.093] | [-0.149,-0.092] |
| Self-Rated Health |  |  |  |  |  |  |
| Very Good | -0.003 | -0.003 | 0.024 | 0.024 | 0.018 | 0.017 |
|  | [-0.035,0.028] | [-0.034,0.028] | [-0.024,0.073] | [-0.024,0.073] | [-0.026,0.062] | [-0.026,0.061] |
| Good | -0.032 | -0.031 | -0.005 | -0.005 | 0.008 | 0.005 |
|  | [-0.079,0.016] | [-0.078,0.016] | [-0.050,0.041] | [-0.050,0.041] | [-0.045,0.061] | [-0.047,0.058] |
| Fair | -0.098*** | -0.098** | -0.066* | -0.066* | -0.019 | -0.021 |
|  | [-0.155,-0.042] | [-0.155,-0.041] | [-0.122,-0.010] | [-0.122,-0.011] | [-0.078,0.040] | [-0.079,0.037] |
| Poor | -0.193*** | -0.193*** | -0.165** | -0.165** | -0.024 | -0.025 |
|  | [-0.281,-0.104] | [-0.281,-0.104] | [-0.268,-0.063] | [-0.268,-0.063] | [-0.131,0.082] | [-0.131,0.081] |
| NA | -0.181 | -0.179 | 0.530*** | 0.527*** | 0.144 | 0.123 |
|  | [-0.981,0.619] | [-0.979,0.620] | [0.311,0.748] | [0.309,0.746] | [-0.168,0.457] | [-0.198,0.444] |
| Non-Metropolitan | -0.042 | -0.042 | -0.019 | -0.019 | -0.027 | -0.027 |
|  | [-0.099,0.015] | [-0.099,0.015] | [-0.072,0.034] | [-0.072,0.035] | [-0.088,0.033] | [-0.086,0.031] |
| Variance of Constant | 0.364*** | 0.364*** | 0.264*** | 0.264*** | 0.335*** | 0.335*** |
|  | [0.340,0.388] | [0.340,0.388] | [0.247,0.281] | [0.247,0.280] | [0.312,0.358] | [0.311,0.358] |
| Variance of Cognition | 0.396*** | 0.396*** | 0.562*** | 0.562*** | 0.398*** | 0.396*** |
|  | [0.382,0.409] | [0.382,0.409] | [0.539,0.586] | [0.539,0.586] | [0.380,0.416] | [0.378,0.414] |
| Observations | 31038 | 31038 | 31187 | 31187 | 31038 | 31038 |
| 95% CIs in brackets  **p*<0.05, ***p*<0.01, ****p*<0.001 | | | | | | |
